# Inborn errors of type I IFN immunity in patients with life-threatening COVID-19

**DOI:** 10.1126/science.abd4570

**Published:** 2020-09-24

**Authors:** Qian Zhang, Paul Bastard, Zhiyong Liu, Jérémie Le Pen, Marcela Moncada-Velez, Jie Chen, Masato Ogishi, Ira K. D. Sabli, Stephanie Hodeib, Cecilia Korol, Jérémie Rosain, Kaya Bilguvar, Junqiang Ye, Alexandre Bolze, Benedetta Bigio, Rui Yang, Andrés Augusto Arias, Qinhua Zhou, Yu Zhang, Fanny Onodi, Sarantis Korniotis, Léa Karpf, Quentin Philippot, Marwa Chbihi, Lucie Bonnet-Madin, Karim Dorgham, Nikaïa Smith, William M. Schneider, Brandon S. Razooky, Hans-Heinrich Hoffmann, Eleftherios Michailidis, Leen Moens, Ji Eun Han, Lazaro Lorenzo, Lucy Bizien, Philip Meade, Anna-Lena Neehus, Aileen Camille Ugurbil, Aurélien Corneau, Gaspard Kerner, Peng Zhang, Franck Rapaport, Yoann Seeleuthner, Jeremy Manry, Cecile Masson, Yohann Schmitt, Agatha Schlüter, Tom Le Voyer, Taushif Khan, Juan Li, Jacques Fellay, Lucie Roussel, Mohammad Shahrooei, Mohammed F. Alosaimi, Davood Mansouri, Haya Al-Saud, Fahd Al-Mulla, Feras Almourfi, Saleh Zaid Al-Muhsen, Fahad Alsohime, Saeed Al Turki, Rana Hasanato, Diederik van de Beek, Andrea Biondi, Laura Rachele Bettini, Mariella D’Angio’, Paolo Bonfanti, Luisa Imberti, Alessandra Sottini, Simone Paghera, Eugenia Quiros-Roldan, Camillo Rossi, Andrew J. Oler, Miranda F. Tompkins, Camille Alba, Isabelle Vandernoot, Jean-Christophe Goffard, Guillaume Smits, Isabelle Migeotte, Filomeen Haerynck, Pere Soler-Palacin, Andrea Martin-Nalda, Roger Colobran, Pierre-Emmanuel Morange, Sevgi Keles, Fatma Çölkesen, Tayfun Ozcelik, Kadriye Kart Yasar, Sevtap Senoglu, Şemsi Nur Karabela, Carlos Rodríguez-Gallego, Giuseppe Novelli, Sami Hraiech, Yacine Tandjaoui-Lambiotte, Xavier Duval, Cédric Laouénan, COVID-STORM Clinicians, COVID Clinicians, Imagine COVID Group, French COVID Cohort Study Group, CoV-Contact Cohort, Amsterdam UMC Covid-19 Biobank, COVID Human Genetic Effort, NIAID-USUHS/TAGC COVID Immunity Group, Andrew L. Snow, Clifton L. Dalgard, Joshua D. Milner, Donald C. Vinh, Trine H. Mogensen, Nico Marr, András N. Spaan, Bertrand Boisson, Stéphanie Boisson-Dupuis, Jacinta Bustamante, Anne Puel, Michael J. Ciancanelli, Isabelle Meyts, Tom Maniatis, Vassili Soumelis, Ali Amara, Michel Nussenzweig, Adolfo García-Sastre, Florian Krammer, Aurora Pujol, Darragh Duffy, Richard P. Lifton, Shen-Ying Zhang, Guy Gorochov, Vivien Béziat, Emmanuelle Jouanguy, Vanessa Sancho-Shimizu, Charles M. Rice, Laurent Abel, Luigi D. Notarangelo, Aurélie Cobat, Helen C. Su, Jean-Laurent Casanova

**Affiliations:** 1 St. Giles Laboratory of Human Genetics of Infectious Diseases, Rockefeller Branch, The Rockefeller University, New York, NY, USA.; 2 Laboratory of Human Genetics of Infectious Diseases, Necker Branch, INSERM U1163, Necker Hospital for Sick Children, Paris, France.; 3 University of Paris, Imagine Institute, Paris, France.; 4 Laboratory of Virology and Infectious Disease, The Rockefeller University, New York, NY, USA.; 5 Department of Paediatric Infectious Diseases & Virology, Imperial College London, London, UK.; 6 Yale Center for Genome Analysis and Department of Genetics, Yale School of Medicine, New Haven, CT, USA.; 7 Zukerman Mind Brain Behavior Institute, Columbia University, New York, NY, USA.; 8 Helix, San Mateo, CA, USA.; 9 Primary Immunodeficiencies Group, University of Antioquia UdeA, Medellin, Colombia.; 10 School of Microbiology, University of Antioquia UdeA, Medellin, Colombia.; 11 Laboratory of Clinical Immunology and Microbiology, Division of Intramural Research, NIAID, NIH, Bethesda, MD, USA.; 12 NIAID Clinical Genomics Program, NIH, Bethesda, MD, USA.; 13 Université de Paris, Institut de Recherche Saint-Louis, INSERM U976, Hôpital Saint-Louis, Paris, France.; 14 Laboratory of Genomes & Cell Biology of Disease, INSERM U944, CNRS UMR 7212, Université de Paris, Institut de Recherche Saint-Louis, Hôpital Saint-Louis, Paris, France.; 15 Sorbonne Université, Inserm, Centre d’Immunologie et des Maladies Infectieuses–Paris (CIMI PARIS), Assistance Publique-Hôpitaux de Paris (AP-HP) Hôpital Pitié-Salpêtrière, Paris, France.; 16 Translational Immunology Lab, Institut Pasteur, Paris, France.; 17 Laboratory for Inborn Errors of Immunity, Department of Microbiology, Immunology and Transplantation, Department of Pediatrics, University Hospitals Leuven, KU Leuven, Leuven, Belgium.; 18 Department of Microbiology, Icahn School of Medicine at Mount Sinai, New York, NY, USA.; 19 Sorbonne Université, UMS037, PASS, Plateforme de Cytométrie de la Pitié-Salpêtrière CyPS, Paris, France.; 20 Bioinformatics Platform, Structure Fédérative de Recherche Necker, INSERM UMR1163, Université de Paris, Imagine Institute, Paris, France.; 21 Neurometabolic Diseases Laboratory, IDIBELL-Hospital Duran i Reynals, CIBERER U759, and Catalan Institution of Research and Advanced Studies (ICREA), Barcelona, Spain.; 22 Department of Immunology, Research Branch, Sidra Medicine, Doha, Qatar.; 23 School of Life sciences, Ecole Polytechnique Fédérale de Lausanne, Lausanne, Switzerland.; 24 Precision Medicine Unit, Lausanne University Hospital and University of Lausanne, Lausanne, Switzerland.; 25 Swiss Institue of Bioinformatics, Lausanne, Switzerland.; 26 Infectious Disease Susceptibility Program, Research Institute, McGill University Health Centre, Montréal, Québec, Canada.; 27 Specialized Immunology Laboratory of Dr. Shahrooei, Sina Medical Complex, Ahvaz, Iran.; 28 Department of Microbiology and Immunology, Clinical and Diagnostic Immunology, KU Leuven, Leuven, Belgium.; 29 Department of Pathology and Laboratory Medicine, College of Medicine, King Saud University, Riyadh, Saudi Arabia.; 30 Department of Clinical Immunology and Infectious Diseases, National Research Institute of Tuberculosis and Lung Diseases, Shahid Beheshti University of Medical Sciences, Tehran, Iran.; 31 The Clinical Tuberculosis and Epidemiology Research Center, National Research Institute of, Tuberculosis and Lung Diseases (NRITLD), Masih Daneshvari Hospital, Shahid Beheshti, University of Medical Sciences, Tehran, Iran.; 32 Pediatric Respiratory Diseases Research Center, National Research Institute of Tuberculosis and Lung Diseases, Shahid Beheshti, Iran.; 33 National Center of Genomics Technology, King Abdulaziz City for Science and Technology, Riyadh, Saudi Arabia.; 34 Dasman Diabetes Institute, Department of Genetics and Bioinformatics, Kuwait.; 35 Immunology Research Laboratory, Department of Pediatrics, College of Medicine and King Saud University Medical City, King Saud University, Riyadh, Saudi Arabia.; 36 Translational Pathology, Department of Pathology and Laboratory Medicine, King Abdulaziz Medical City, Misery of National Guard Health Affairs, Riyadh, Saudi Arabia.; 37 Cancer & Blood Research, King Abdullah International Medical Research Center, Riyadh, Saudi Arabia.; 38 Amsterdam UMC, Department of Neurology, Amsterdam Neuroscience, Amsterdam, Netherlands.; 39 Pediatric Departement and Centro Tettamanti-European Reference Network PaedCan, EuroBloodNet, MetabERN-University of Milano-Bicocca-Fondazione MBBM-Ospedale, San Gerardo, Monza, Italy.; 40 Department of Infectious Diseases, San Gerardo Hospital–University of Milano-Bicocca, Monza, Italy.; 41 CREA Laboratory, Diagnostic Laboratory, ASST Spedali Civili di Brescia, Brescia, Italy.; 42 Department of Infectious and Tropical Diseases, University of Brescia and ASST Spedali di Brescia, Brescia, Italy.; 43 Chief Medical Officer, ASST Spedali Civili di Brescia, Brescia, Italy.; 44 Bioinformatics and Computational Biosciences Branch, Office of Cyber Infrastructure and Computational Biology, NIAID, NIH, Bethesda, MD, USA.; 45 PRIMER, Uniformed Services University of the Health Sciences, Bethesda, MD, USA.; 46 Center of Human Genetics, Hôpital Erasme, Université Libre de Bruxelles, Brussels, Belgium.; 47 Department of Internal Medicine, Hôpital Erasme, Université Libre de Bruxelles, Brussels, Belgium.; 48 Fonds de la Recherche Scientifique (FNRS) and Center of Human Genetics, Hôpital Erasme, Université Libre de Bruxelles, Brussels, Belgium.; 49 Department of Paediatric Immunology and Pulmonology, Centre for Primary Immunodeficiency Ghent (CPIG), PID Research Lab, Jeffrey Modell Diagnosis and Research Centre, Ghent University Hospital, Ghent, Belgium.; 50 Pediatric Infectious Diseases and Immunodeficiencies Unit, Hospital Universitari Vall d’Hebron, Vall d’Hebron Research Institute, Vall d’Hebron Barcelona Hospital Campus, Universitat Autònoma de Barcelona (UAB), Barcelona, Catalonia, Spain.; 51 Immunology Division, Genetics Department, Hospital Universitari Vall d’Hebron, Vall d’Hebron Research Institute, Vall d’Hebron Barcelona Hospital Campus, UAB, Barcelona, Catalonia, Spain.; 52 Aix Marseille Univ, INSERM, INRAE, C2VN, CHU Timone, Marseille, France.; 53 Necmettin Erbakan University, Meram Medical Faculty, Division of Pediatric Allergy and Immunology, Konya, Turkey.; 54 Department of Infectious Diseases and Clinical Microbiology, Konya Training and Research Hospital, Konya, Turkey.; 55 Department of Molecular Biology and Genetics, Bilkent University, Bilkent-Ankara, Turkey.; 56 Departments of Infectious Diseases and Clinical Microbiology, Bakirkoy Dr. Sadi Konuk Training and Research Hospital, University of Health Sciences, Istanbul, Turkey.; 57 Department of Immunology, Hospital Universitario de G.C. Dr. Negrín, Canarian Health System, Las Palmas de Gran Canaria, Spain.; 58 University Fernando Pessoa Canarias, Las Palmas de Gran Canaria, Spain.; 59 Department of Biomedicine and Prevention, University of Rome “Tor Vergata,” Rome, Italy.; 60 Intensive Care Unit, AP-HM, Marseille, France.; 61 Avicenne Hospital Intensive Care Unit, APHP, Bobigny, INSERM U1272 Hypoxia & Lung, Paris, France.; 62 PH Réanimation CHU Avicenne, Bobigny, INSERM U1272 Hypoxie & Poumon, Paris, France.; 63 Université de Paris, IAME UMR-S 1137, INSERM, Paris, France.; 64 Inserm CIC 1425, Paris, France.; 65 AP-HP, Département Epidémiologie Biostatistiques et Recherche Clinique, Hôpital Bichat, Paris, France.; 66 Department of Pharmacology & Molecular Therapeutics, Uniformed Services University of the Health Sciences, Bethesda, MD, USA.; 67 Department of Anatomy, Physiology & Genetics, Uniformed Services University of the Health Sciences, Bethesda, MD, USA.; 68 Division of Pediatric Allergy, Immunology and Rheumatology, Columbia University, New York, USA.; 69 Department of Infectious Diseases, Aarhus University Hospital, Skejby, Denmark.; 70 Department of Biomedicine, Aarhus University, Aarhus, Denmark.; 71 College of Health and Life Sciences, Hamad Bin Khalifa University, Doha, Qatar.; 72 Department of Medical Microbiology, Utrecht UMC, Utrecht, Netherlands.; 73 Study Center for Primary Immunodeficiencies, Necker Hospital for Sick Children, Paris, France.; 74 Turnstone Biologics, New York, NY, USA.; 75 Department of Pediatrics, University Hospitals Leuven, KU Leuven, Leuven, Belgium.; 76 New York Genome Center, New York, NY, USA.; 77 AP-HP, Hôpital Saint-Louis, Laboratoire d’Immunologie, Paris, France.; 78 Laboratory of Molecular Immunology, Rockefeller University, New York, NY, USA.; 79 Howard Hughes Medical Institute, New York, NY, USA.; 80 Department of Medicine, Division of Infectious Diseases, Icahn School of Medicine at Mount Sinai, New York, NY, USA.; 81 Global Health and Emerging Pathogens Institute, Icahn School of Medicine at Mount Sinai, New York, NY, USA.; 82 The Tisch Cancer Institute, Icahn School of Medicine at Mount Sinai, New York, NY, USA.; 83 Laboratory of Genetics and Genomics, The Rockefeller University, New York, NY, USA.; 84 Department of Genetics, Yale University School of Medicine, New Haven, CT, USA.; 85 Yale Center for Genome Analysis, Yale School of Medicine, New Haven, CT, USA.; 86 Pediatric Hematology and Immunology Unit, Necker Hospital for Sick Children, AP-HP, Paris, France.

## Abstract

Clinical outcome upon infection with severe acute respiratory syndrome coronavirus 2 (SARS-CoV-2) ranges from silent infection to lethal coronavirus disease 2019 (COVID-19). We have found an enrichment in rare variants predicted to be loss-of-function (LOF) at the 13 human loci known to govern Toll-like receptor 3 (TLR3)– and interferon regulatory factor 7 (IRF7)–dependent type I interferon (IFN) immunity to influenza virus in 659 patients with life-threatening COVID-19 pneumonia relative to 534 subjects with asymptomatic or benign infection. By testing these and other rare variants at these 13 loci, we experimentally defined LOF variants underlying autosomal-recessive or autosomal-dominant deficiencies in 23 patients (3.5%) 17 to 77 years of age. We show that human fibroblasts with mutations affecting this circuit are vulnerable to SARS-CoV-2. Inborn errors of TLR3- and IRF7-dependent type I IFN immunity can underlie life-threatening COVID-19 pneumonia in patients with no prior severe infection.

## The genetics underlying severe COVID-19

The immune system is complex and involves many genes, including those that encode cytokines known as interferons (IFNs). Individuals that lack specific IFNs can be more susceptible to infectious diseases. Furthermore, the autoantibody system dampens IFN response to prevent damage from pathogen-induced inflammation. Two studies now examine the likelihood that genetics affects the risk of severe coronavirus disease 2019 (COVID-19) through components of this system (see the Perspective by Beck and Aksentijevich). Q. Zhang *et al.* used a candidate gene approach and identified patients with severe COVID-19 who have mutations in genes involved in the regulation of type I and III IFN immunity. They found enrichment of these genes in patients and conclude that genetics may determine the clinical course of the infection. Bastard *et al.* identified individuals with high titers of neutralizing autoantibodies against type I IFN-α2 and IFN-ω in about 10% of patients with severe COVID-19 pneumonia. These autoantibodies were not found either in infected people who were asymptomatic or had milder phenotype or in healthy individuals. Together, these studies identify a means by which individuals at highest risk of life-threatening COVID-19 can be identified.

*Science*, this issue p. eabd4570, p. eabd4585; see also p. 404

## Abstract (structured)

**INTRODUCTION:**

Clinical outcomes of human severe acute respiratory syndrome coronavirus 2 (SARS-CoV-2) infection range from silent infection to lethal coronavirus disease 2019 (COVID-19). Epidemiological studies have identified three risk factors for severe disease: being male, being elderly, and having other medical conditions. However, interindividual clinical variability remains huge in each demographic category. Discovering the root cause and detailed molecular, cellular, and tissue- and body-level mechanisms underlying life-threatening COVID-19 is of the utmost biological and medical importance.

**RATIONALE:**

We established the COVID Human Genetic Effort (www.covidhge.com) to test the general hypothesis that life-threatening COVID-19 in some or most patients may be caused by monogenic inborn errors of immunity to SARS-CoV-2 with incomplete or complete penetrance. We sequenced the exome or genome of 659 patients of various ancestries with life-threatening COVID-19 pneumonia and 534 subjects with asymptomatic or benign infection. We tested the specific hypothesis that inborn errors of Toll-like receptor 3 (TLR3)– and interferon regulatory factor 7 (IRF7)–dependent type I interferon (IFN) immunity that underlie life-threatening influenza pneumonia also underlie life-threatening COVID-19 pneumonia. We considered three loci identified as mutated in patients with life-threatening influenza: *TLR3*, *IRF7*, and *IRF9*. We also considered 10 loci mutated in patients with other viral illnesses but directly connected to the three core genes conferring influenza susceptibility: *TICAM1/TRIF*, *UNC93B1*, *TRAF3*,* TBK1*,* IRF3*, and *NEMO/IKBKG* from the TLR3-dependent type I IFN induction pathway, and *IFNAR1*, *IFNAR2*, *STAT1*, and *STAT2* from the IRF7- and IRF9-dependent type I IFN amplification pathway. Finally, we considered various modes of inheritance at these 13 loci.

**RESULTS:**

We found an enrichment in variants predicted to be loss-of-function (pLOF), with a minor allele frequency <0.001, at the 13 candidate loci in the 659 patients with life-threatening COVID-19 pneumonia relative to the 534 subjects with asymptomatic or benign infection (*P *= 0.01). Experimental tests for all 118 rare nonsynonymous variants (including both pLOF and other variants) of these 13 genes found in patients with critical disease identified 23 patients (3.5%), aged 17 to 77 years, carrying 24 deleterious variants of eight genes. These variants underlie autosomal-recessive (AR) deficiencies (*IRF7* and *IFNAR1*) and autosomal-dominant (AD) deficiencies (*TLR3*,* UNC93B1*,* TICAM1*,* TBK1*,* IRF3*,* IRF7*,* IFNAR1*, and *IFNAR2*) in four and 19 patients, respectively. These patients had never been hospitalized for other life-threatening viral illness. Plasmacytoid dendritic cells from IRF7-deficient patients produced no type I IFN on infection with SARS-CoV-2, and TLR3^−/−^, TLR3^+/−^, IRF7^−/−^, and IFNAR1^−/−^ fibroblasts were susceptible to SARS-CoV-2 infection in vitro.

**CONCLUSION:**

At least 3.5% of patients with life-threatening COVID-19 pneumonia had known (AR IRF7 and IFNAR1 deficiencies or AD TLR3, TICAM1, TBK1, and IRF3 deficiencies) or new (AD UNC93B1, IRF7, IFNAR1, and IFNAR2 deficiencies) genetic defects at eight of the 13 candidate loci involved in the TLR3- and IRF7-dependent induction and amplification of type I IFNs. This discovery reveals essential roles for both the double-stranded RNA sensor TLR3 and type I IFN cell-intrinsic immunity in the control of SARS-CoV-2 infection. Type I IFN administration may be of therapeutic benefit in selected patients, at least early in the course of SARS-CoV-2 infection.

## Teaser

A large immunological and genomics study of COVID-19 patients reveals excess mutations in the type I IFN pathway.

## Graphical abstract

**Figure:**
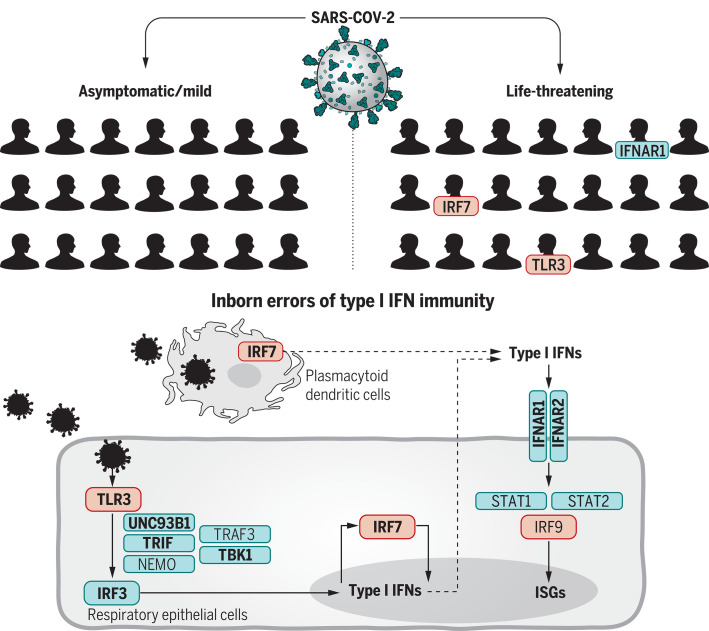
Inborn errors of TLR3- and IRF7-dependent type I IFN production and amplification underlie life-threatening COVID-19 pneumonia. Molecules in red are encoded by core genes, deleterious variants of which underlie critical influenza pneumonia with incomplete penetrance, and deleterious variants of genes encoding biochemically related molecules in blue underlie other viral illnesses. Molecules represented in bold are encoded by genes with variants that also underlie critical COVID-19 pneumonia.

Severe acute respiratory syndrome coronavirus 2 (SARS-CoV-2) has already claimed at least 1 million lives, has been detected in at least 20 million people, and has probably infected at least another 200 million. The clinical manifestations range from silent infection to lethal disease, with an infection fatality rate of 0.1 to 0.9%. Three epidemiological factors increase the risk of severity: (i) increasing age, decade by decade, after the age of 50, (ii) being male, and (iii) having various underlying medical conditions (*1*). However, even taking these factors into account, there is immense interindividual clinical variability in each demographic category considered. Following on from our human genetic studies of other severe infectious diseases (*2*, *3*), we established the COVID Human Genetic Effort (https://www.covidhge.com) to test the general hypothesis that in some patients, life-threatening coronavirus disease 2019 (COVID-19) may be caused by monogenic inborn errors of immunity to SARS-CoV-2 with incomplete or complete penetrance (*4*). We enrolled 659 patients (74.5% men and 25.5% women, 13.9% of whom died) of various ancestries between 1 month and 99 years of age (Fig. 1A). These patients were hospitalized for life-threatening pneumonia caused by SARS-CoV-2 (critical COVID-19). We sequenced their whole genome (*N* = 364) or exome (*N* = 295), and principal component analysis (PCA) on these data confirmed their ancestries (Fig. 1B).

**Fig. 1:**
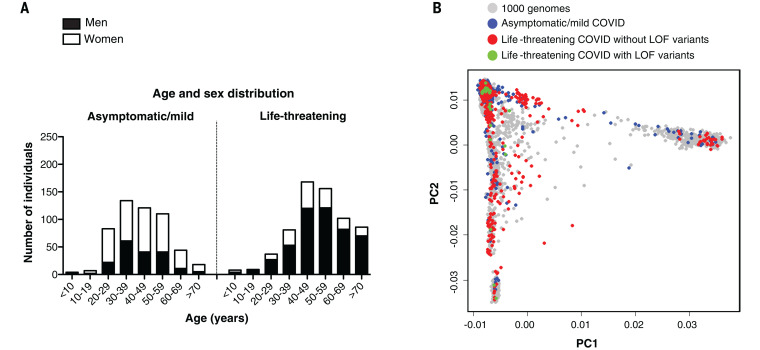
Demographic and genetic data for the COVID-19 cohort. (**A**) Age and sex distribution of patients with life-threatening COVID-19. (**B**) PCA of patient (with or without LOF variants in the 13 candidate genes) and control cohorts (patients with mild or asymptomatic disease and individuals from the 1000 Genomes Project).

## Candidate variants at 13 human loci that govern immunity to influenza virus

**Fig. 2:**
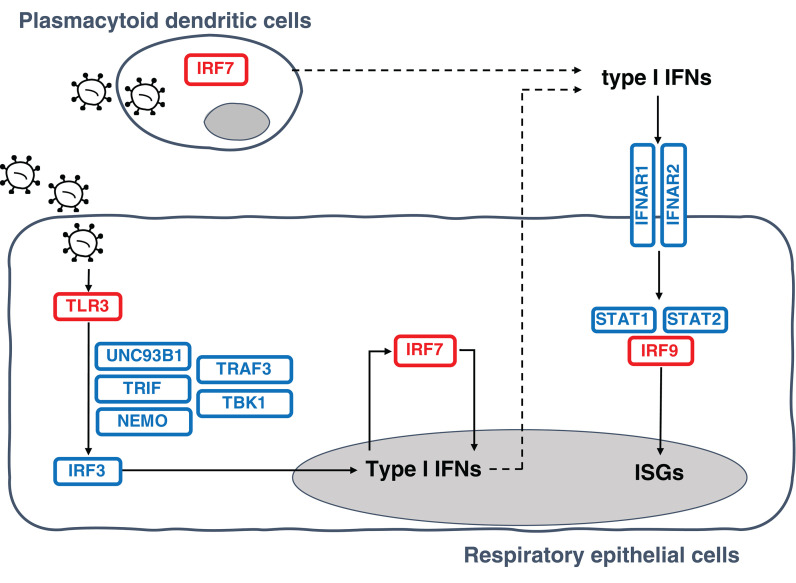
Illustration of TLR3- and IRF7-dependent type I IFN production and amplification circuit. Molecules in red are encoded by core genes, deleterious variants of which underlie critical influenza pneumonia with incomplete penetrance; deleterious variants of genes encoding biochemically related molecules in blue underlie other viral illnesses. Type I IFNs also induce themselves. ISGs, interferon-stimulated genes.

We first tested the specific hypothesis that inborn errors of Toll-like receptor 3 (TLR3)– and interferon regulatory factor 7 (IRF7)–dependent type I interferon (IFN) immunity, which underlie life-threatening influenza pneumonia, may also underlie life-threatening COVID-19 pneumonia (*5*) (Fig. 2). We considered three loci previously shown to be mutated in patients with critical influenza pneumonia: *TLR3* (*6*), *IRF7* (*7*), and *IRF9* (*8*). We also considered 10 loci mutated in patients with other viral illnesses but directly connected to the three core genes conferring influenza susceptibility: *TICAM1/TRIF* (*9*), *UNC93B1* (*10*), *TRAF3* (*11*), *TBK1* (*12*), *IRF3* (*13*), and *NEMO/IKBKG* (*14*) in the TLR3-dependent type I IFN induction pathway, and *IFNAR1* (*15*), *IFNAR2* (*16*), *STAT1* (*17*), and *STAT2* (*18*) in the IRF7- and IRF9-dependent type I IFN amplification pathway. We collected both monoallelic and biallelic nonsynonymous variants with a minor allele frequency (MAF) <0.001 at all 13 loci. Twelve of the 13 candidate loci are autosomal, whereas *NEMO* is X-linked. For the latter gene, we considered only a recessive model (*19*). Autosomal-dominant (AD) inheritance has not been proven for six of the 12 autosomal loci (*UNC93B1*, *IRF7*, *IFNAR1*, *IFNAR2*, *STAT2*, and *IRF9*). Nevertheless, we considered heterozygous variants because none of the patients enrolled had been hospitalized for critical viral infections before COVID-19, raising the possibility that any underlying genetic defects that they might have display a lower penetrance for influenza and other viral illnesses than for COVID-19, which is triggered by a more virulent virus.

## Enrichment of variants predicted to be LOF at the influenza susceptibility loci

We found four unrelated patients with biallelic variants of *IRF7* or *IFNAR1* (Table 1 and table S1). We also found 113 patients carrying 113 monoallelic variants at 12 loci: *TLR3* (*N* = 7 patients/7 variants), *UNC93B1* (*N* = 10/9), *TICAM1* (*N* = 17/15), *TRAF3* (*N* = 6/6), *TBK1* (*N* = 12/11), *IRF3* (*N* = 5/5), *IRF7* (*N* = 20/13), *IFNAR1* (*N* = 14/13), *IFNAR2* (*N* = 17/15), *STAT1* (*N* = 4/4), *STAT2* (*N* = 11/11), and *IRF9* (*N* = 4/4). We detected no copy number variation for these 13 genes. Unexpectedly, one of these variants has been reported in patients with life-threatening influenza pneumonia (*TLR3* p.Pro554Ser) (*6*, *20*) and another was shown to be both deleterious and dominant-negative (*IFNAR1* p.Pro335del) (*21*). Nine of the 118 biallelic or monoallelic variants were predicted to be LOF (pLOF), whereas the remaining 109 were missense or in-frame indels (table S1). In a sample of 534 controls with asymptomatic or mild SARS-CoV-2 infection, we found only one heterozygous pLOF variation with a MAF <0.001 at the 13 loci (*IRF7* p.Leu99fs). A PCA-adjusted burden test on the 12 autosomal loci revealed significant enrichment in pLOF variants in patients relative to controls [*P* = 0.01; odds ratio (OR) = 8.28; 95% confidence interval (CI) = 1.04 to 65.64] under an AD mode of inheritance. The same analysis performed on synonymous variants with a MAF <0.001 was not significant (*P* = 0.19), indicating that our ethnicity-adjusted burden test was well calibrated.

**Table 1:** Disease-causing variants identified in patients with life-threatening COVID-19.

**Gene**	**Inheritance**	**Genetic form**	**Genotype**	**Gender**	**Age** [**years**]	**Ancestry/residence**	**Outcome**
*TLR3*	AD	Known	p.Ser339fs/WT	M	40	Spain	Survived
*TLR3*	AD	Known	p.Pro554Ser/WT	M	68	Italy	Survived
*TLR3*	AD	Known	p.Trp769*/WT	M	77	Italy	Survived
*TLR3*	AD	Known	p.Met870Val/WT	M	56	Colombia/Spain	Survived
*UNC93B1*	AD	New	p.Glu96*/WT	M	48	Venezuela/Spain	Survived
*TICAM1*	AD	Known	p.Thr4Ile/WT	M	49	Italy	Survived
*TICAM1*	AD	Known	p.Ser60Cys/WT	F	61	Vietnam/France	Survived
*TICAM1*	AD	Known	p.Gln392Lys/WT	F	71	Italy	Deceased
*TBK1*	AD	Known	p.Phe24Ser/WT	F	46	Venezuela/Spain	Survived
*TBK1*	AD	Known	p.Arg308*/WT	M	17	Turkey	Survived
*IRF3*	AD	Known	p.Glu49del/WT	F	23	Bolivia/Spain	Survived
*IRF3*	AD	Known	p.Asn146Lys/WT	F	60	Italy	Survived
*IRF7*	AR	Known	p.Pro364fs/p.Pro364fs	F	49	Italy/Belgium	Survived
*IRF7*	AR	Known	p.Met371Val/p.Asp117Asn	M	50	Turkey	Survived
*IRF7*	AD	New	p.Arg7fs/WT	M	60	Italy	Survived
*IRF7*	AD	New	p.Gln185*/WT	M	44	France	Survived
*IRF7*	AD	New	p.Pro246fs/WT	M	41	Spain	Survived
*IRF7*	AD	New	p.Arg369Gln/WT	M	69	Italy	Survived
*IRF7*	AD	New	p.Phe95Ser/WT	M	37	Turkey	Survived
*IFNAR1*	AR	Known	p.Trp73Cys/Trp73Cys	M	38	Turkey	Survived
*IFNAR1*	AR	Known	p.Ser422Arg/Ser422Arg	M	26	Pakistan/Saudi Arabia	Deceased
*IFNAR1*	AD	New	p.Pro335del/WT	F	23	China/Italy	Survived
*IFNAR2*	AD	New	p.Glu140fs/WT	F	54	Belgium	Survived

## Experimentally deleterious alleles at the influenza susceptibility loci in 3.5% of patients

**Fig. 3:**
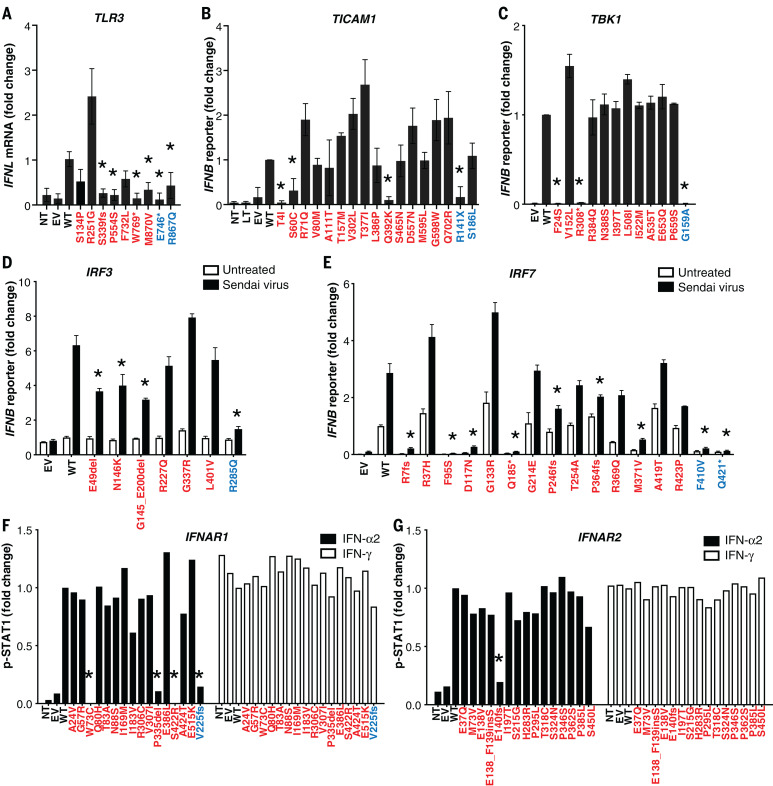
Impact of *TLR3*, *TICAM1*, *TBK1*, *IRF3*, *IRF7*, *IFNAR1*, and *IFNAR2* variants on type I IFN signaling. (**A**) TLR3-deficient P2.1 fibrosarcoma cells were stably transfected with plasmids expressing WT or mutant forms of *TLR3*, and *IFNL1* mRNA levels were determined by reverse transcription quantitative PCR. *IFNL1* mRNA levels were expressed relative to the housekeeping gene *GUS* and then normalized. *IFNL1* was undetectable in unstimulated cells. The differences between variants and WT were tested using one-way ANOVA (*P < 0.05). (**B**) TICAM1-deficient SV40-Fib cells were transiently transfected with WT or mutant forms of *TICAM1*, together with an IFN-β luciferase reporter and a constitutively expressed reporter. Normalized luciferase induction was measured 24 hours after transfection. The differences between variants and WT were tested using one-way ANOVA (*P < 0.05). (**C**) HEK293T cells were transiently transfected with WT and mutant forms of *TBK1*, together with an IFN-β luciferase reporter and a constitutively expressed reporter. Normalized luciferase activity was measured 24 hours after transfection. The differences between variants and WT were tested using one-way ANOVA (*P < 0.05). (**D**) IRF3-deficient HEK293T cells were transiently transfected with WT and mutant forms of *IRF3*, together with an IFN-β luciferase reporter and a constitutively expressed reporter. Cells were either left untreated or infected with Sendai virus for 24 hours before the normalized measurement of luciferase activity. The differences between variants and WT were evaluated using two-way ANOVA (*P < 0.05). (**E**) HEK293T cells were transiently transfected with WT and mutant forms of *IRF7*, together with an IFN-β luciferase reporter and a constitutively expressed reporter. Cells were either left untreated or infected with Sendai virus for 24 hours before the normalized measurement of luciferase activity. The differences between variants and WT were tested using two-way ANOVA (*P < 0.05). (**F** and **G**) IFNAR1- or IFNAR2-deficient SV40-Fib cells were transiently transfected with WT or mutant forms of *IFNAR1* for 36 hours, and either left untreated or stimulated with IFN-α2 or IFN-γ. Fluorescence-activated cell sorting (FACS) staining with anti-p-STAT1 antibody and the *z*-score of the MFI were assessed. Asterisks indicate variants with MFI <50% of WT. Variants in red were identified in COVID-19 patients. Variants in blue are known deleterious variants and served as negative controls. EV, empty vector; LT, lipofectamine. Three technical repeats were performed for (A) to (E). Means and SD are shown in the columns and horizontal bars when appropriate.

We tested these 118 variants experimentally in ad hoc overexpression systems. We found that 24 variants of eight genes were deleterious (including all the pLOF variants) because they were loss-of-expression, LOF, or severely hypomorphic: *TLR3* (*N* = 4 variants), *UNC93B1* (*N* = 1), *TICAM1* (*N* = 3), *TBK1* (*N* = 2), *IRF3* (*N* = 2), *IRF7* (*N* = 8), *IFNAR1* (*N* = 3), and *IFNAR2* (*N* = 1) (table S1, Fig. 3, and figs. S1 to S8). Consistently, heterozygous LOF variants of *IRF3* and *IRF7* were reported in single patients with life-threatening influenza pneumonia (*22*, *23*). The remaining 94 variants were biochemically neutral. Twenty-three patients carried these 24 deleterious variants, resulting in four autosomal-recessive (AR) deficiencies (homozygosity or compound heterozygosity for *IRF7*; homozygosity for *IFNAR1*) and 19 AD deficiencies. These 23 patients did not carry candidate variants at the other 417 loci known to underlie inborn errors of immunity (table S2) (*24*–*26*). These findings suggest that at least 23 (3.5%) unrelated patients of the 659 patients tested suffered from a deficiency at one of eight loci among the 13 tested: four patients with a known AR disorder (*IRF7* or *IFNAR1*) (*7*, *15*), 11 with a known AD disorder (*TLR3*, *TICAM1*, *TBK1*, or *IRF3*) (*6*, *9*, *12*, *13*, *20*), and eight with a previously unknown AD genetic disorder (*UNC93B1*, *IRF7*, *IFNAR1*, or *IFNAR2*).

## Impaired TLR3- and IRF7-dependent type I immunity in patient cells in vitro

**Fig. 4:**
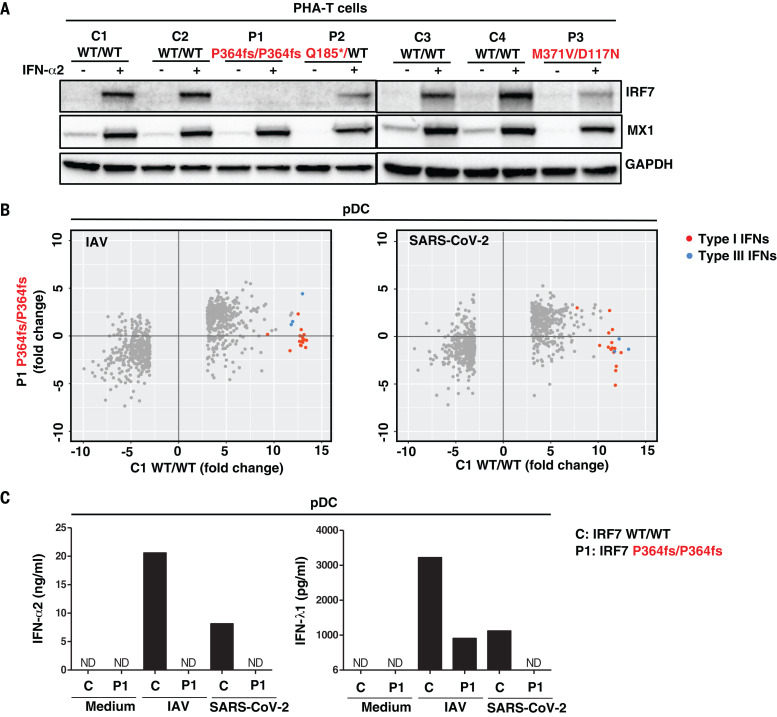
Type I IFN responses in patient cells defective for IRF7. (**A**) Levels of the IRF7 protein in PHA-T cells from two patients with AR IRF7 deficiency (P1 and P3), one patient with AD IRF7 deficiency (P2), and four healthy donors (C1 to C4). Cells were either left untreated or stimulated with IFN-α2 for 24 hours, and protein levels were measured by Western blotting. MX1 was used as a positive control for IFN-α2 treatment. (**B**) pDCs isolated from an AR IRF7-deficient patient (P1) and a healthy donor (C1) were either left untreated or infected with influenza A virus (IAV) or SARS-CoV-2, and RNA-seq was performed. Genes with expression >2.5-fold higher or lower in C1 after infection are plotted as the fold change in expression. Red dots are type I IFN genes; blue dots are type III IFN genes. (**C**) pDCs isolated from healthy donor C and IRF7-deficient patient (P1) were either left untreated (Medium) or infected with IAV or SARS-CoV-2, and the production of IFN-α2 and IFN-λ1 was measured by CBA and ELISA, respectively, on the supernatant. ND, not detected.

**Fig. 5:**
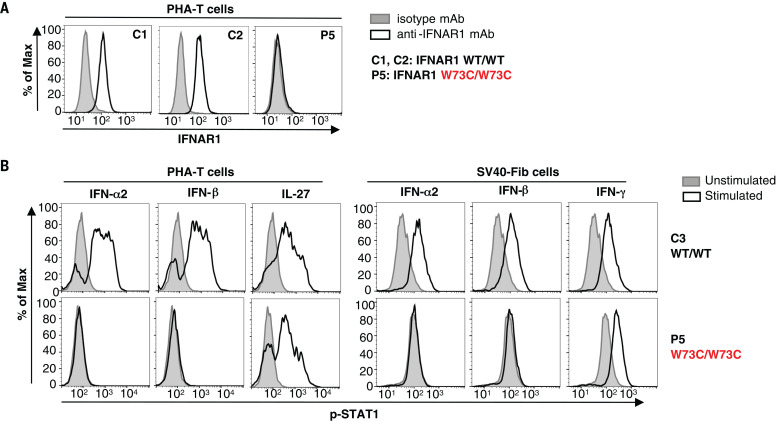
Type I IFN responses in patient cells defective for IFNAR1. (**A**) FACS staining of IFNAR1 on the surface of PHA-T cells from a patient with AR IFNAR1 deficiency (P5) and healthy donors (C1 and C2). (**B**) PHA-T cells and SV40-Fib from a patient with AR IFNAR1 deficiency (P5) and a healthy donor (C3) were stimulated with IFN-α2 or IFN-β, and p-STAT1 levels were determined by FACS. Interleukin-27 stimulation served as a positive control on PHA-T cells, whereas IFN-γ stimulation served as a positive control on SV40-Fib cells.

**Fig. 6:**
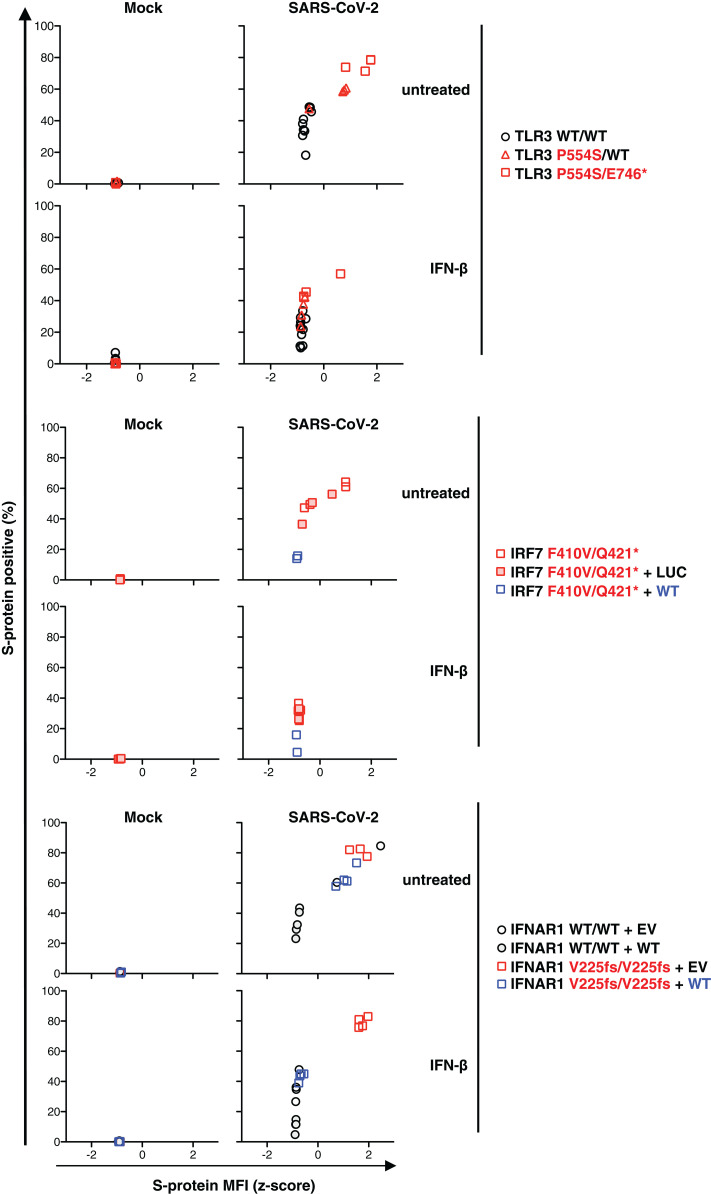
Cell-intrinsic type I IFN response to SARS-CoV-2. SV40-Fib cells of TLR3^−/−^, TLR3^+/−^, IRF7^−/−^, and IRF7^−/−^ SV40-Fib cells rescued with WT IRF7; IFNAR1^−/−^ SV40-Fib cells, and IFNAR1^−/−^ SV40-Fib cells rescued with WT IFNAR1 were transduced with ACE2 and TMPRSS2 and then either left untreated or treated with IFN-β for 4 hours. Cells were then infected with SARS-CoV-2 (MOI = 0.5). After staining, ACE2 and viral S-protein levels were measured by high-content microscopy with gating on ACE2^+^ cells. IRF7-deficient SV40-Fib cells were previously transduced with either WT IRF7 or negative control (Luc). IFNAR1-deficient cells were previously transduced with either WT IFNAR1 or empty vector (EV).

We tested cells from patients with selected genotypes and showed that PHA-driven T cell blasts (PHA-T cells) from patients with AR or AD IRF7 deficiency had low levels of IRF7 expression (Fig. 4A). We then isolated circulating plasmacytoid dendritic cells (pDCs) from a patient with AR IRF7 deficiency (fig. S9A) (*7*). These cells were present in normal proportions (fig. S9B), but they did not produce any detectable type I or III IFNs in response to SARS-CoV-2, as analyzed by cytometric bead array (CBA), enzyme-linked immunosorbent assay (ELISA), and RNA sequencing (RNA-seq) (Fig. 4, B and C). We also showed that PHA-T cells from a patient with AR IFN-α/β receptor 1 (IFNAR1) deficiency had impaired IFNAR1 expression and responses to IFN-α2 or IFN-β, and that the patient’s SV40-transformed fibroblast (SV40-Fib) cells did not respond to IFN-α2 or IFN-β (Fig. 5). We then infected TLR3^−/−^, TLR3^+/−^, IRF7^−/−^ SV40-Fib cells, and IRF7^−/−^ SV40-Fib cells rescued with wild-type (WT) IRF7; IFNAR1^−/−^ SV40-Fib cells, and IFNAR1^−/−^ SV40-Fib cells rescued with WT IFNAR1, all of which were previously transduced with angiotensin-converting enzyme 2 (ACE2) and transmembrane protease, serine 2 (TMPRSS2). SARS-CoV-2 infection levels were higher in mutant cells than in cells from healthy donors, and transduction of WT *IRF7* or *IFNAR1* rescued their defects (Fig. 6). Collectively, these findings showed that AR IRF7 deficiency impaired the production of type I IFN by pDCs stimulated with SARS-CoV-2, whereas AR and AD deficiencies of TLR3 or AR deficiency of IFNAR1 impaired fibroblast-intrinsic type I IFN immunity to SARS-CoV2. They also suggest that heterozygosity for LOF variations at the other five mutated loci also underlie life-threatening COVID-19.

## Impaired production of type I IFNs in patients in vivo

**Fig. 7:**
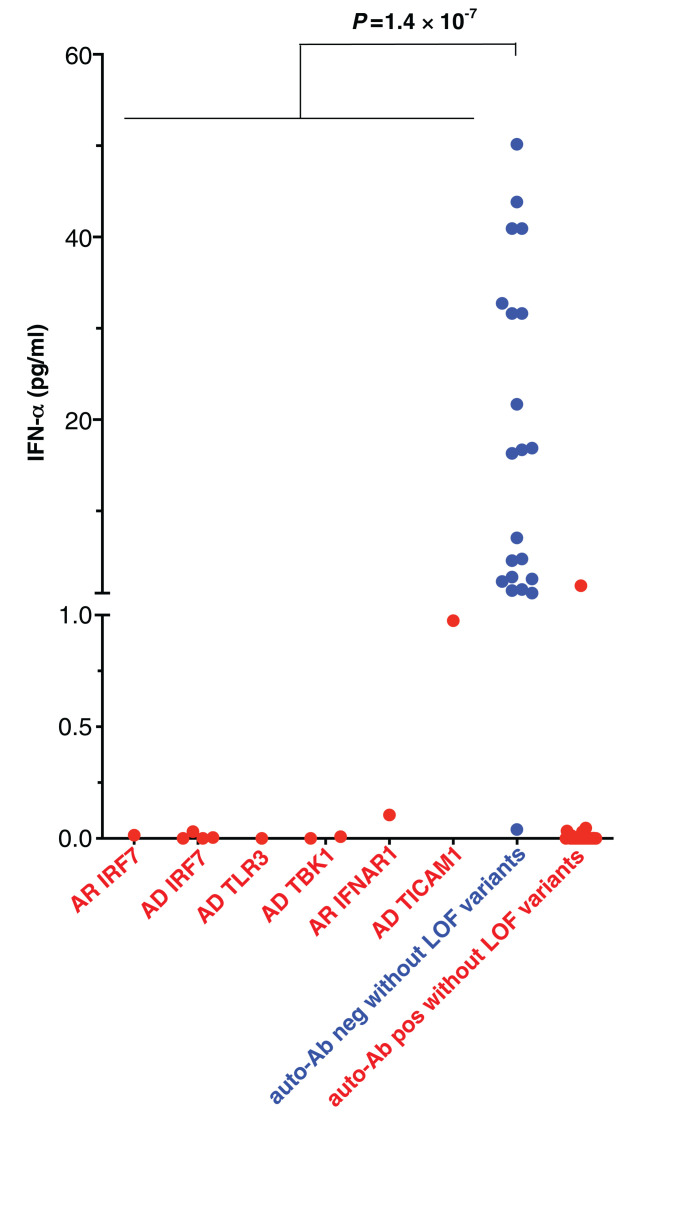
In vivo type I IFN responses to SARS-CoV-2 infections. Plasma levels of 13 IFN-α were measured by Simoa. Auto-Ab(+) without LOF variants indicates COVID-19 patients with neutralizing anti-IFN-α auto-Abs in our accompanying report (*29*). *P* values indicated were evaluated using one-way ANOVA.

We tested whether these genotypes impaired the production of type I IFN in vivo during the course of SARS-CoV-2 infection. We measured the levels of the 13 types of IFN-α in the blood of patients during the acute phase of COVID-19. We found that 10 of the 23 patients with mutations for whom samples were available (one with AR IRF7 deficiency, four with AD IRF7 deficiency, one with AD TLR3 deficiency, two with AD TBK1 deficiency, one with AR IFNAR1 deficiency, and one with AD TICAM1 deficiency) had serum IFN-α levels <1 pg/ml (Fig. 7). By contrast, previously published cohorts of patients hospitalized with unexplained, severe COVID-19 had various serum IFN-α levels, significantly higher than our 10 patients [one-way analysis of variance (ANOVA), *P* = 1.4 × 10^−7^; Fig. 7] (*27*, *28*). Another 29 patients from our cohort displaying auto-antibodies (auto-Abs) against type I IFNs, reported in an accompanying paper, had undetectable levels of serum IFN-α (*29*). Moreover, none of the 23 patients with LOF mutations of the eight genes had detectable auto-Abs against type I IFNs (*29*), strongly suggesting that the two mechanisms of disease are similar but independent. Excluding patients with auto-Abs against type I IFN from the burden test of pLOF variants at the 12 autosomal loci strengthened the association signal (*P* = 0.007; OR = 8.97; 95% CI = 1.13 to 71.09).

## Inborn errors of TLR3- and IRF7-dependent type I immunity underlie critical COVID-19

Collectively, our data suggest that at least 23 of the 659 patients with life-threatening COVID-19 pneumonia studied had known (six disorders) or new (four disorders) genetic defects at eight loci involved in the TLR3- and IRF7-dependent induction and amplification of type I IFNs. This discovery reveals the essential role of both the double-stranded RNA sensor TLR3 and type I IFN cell-intrinsic immunity in the control of SARS-CoV-2 infection in the lungs, consistent with their previously documented roles in pulmonary immunity to influenza virus (*5*–*8*). These genotypes were silent until infection with SARS-CoV-2. The most thought-provoking examples are the AR deficiencies of IRF7 and IFNAR1. AR IRF7 deficiency was diagnosed in two individuals aged 49 and 50 years, and AR IFNAR1 deficiency was diagnosed in two individuals aged 26 and 38 years, and none of the four patients had a prior history of life-threatening infections (Table 1). One patient with IRF7 deficiency was tested and was seropositive for several common viruses, including various influenza A and B viruses (figs. S10 and S11). These genetic defects therefore display incomplete penetrance for influenza respiratory distress and only manifested clinically upon infection with the more virulent SARS-CoV-2.

## Conclusion

The AR form of IFNAR1 deficiency highlights the importance of type I IFN production relative to type III IFN production, which is also impaired by defects of TLR3, IRF7, and IRF9 (*5*). This conclusion is also supported by our accompanying report of neutralizing auto-Abs against type I IFNs, but not type III IFNs, in other patients with life-threatening COVID-19 pneumonia (*29*). Inborn errors of TLR3- and IRF7-dependent type I IFN immunity at eight loci were found in as many as 23 patients (3.5%) of various ages (17 to 77 years) and ancestries (various nationalities from Asia, Europe, Latin America, and the Middle East) and in patients of both sexes (Table 1). Our findings suggest that there may be mutations in other type I IFN–related genes in other patients with life-threatening COVID-19 pneumonia. They also suggest that the administration of type I IFN may be of therapeutic benefit in selected patients, at least early in the course of SARS-CoV-2 infection.

## Methods

### Patients

We included in this study 659 patients with life-threatening COVID-19 pneumonia, defined as patients with pneumonia who developed critical disease, whether pulmonary with mechanical ventilation (CPAP, BIPAP, intubation, hi-flow oxygen), septic shock, or with any other organ damage requiring admission to the intensive care unit. Patients who developed Kawasaki-like syndrome were excluded. The age of the patients ranged from 0.1 to 99 years, with a mean age of 51.8 years (SD 15.9 years), and 25.5% of the patients were female. As controls, we enrolled 534 individuals infected with SARS-CoV-2 based on a positive polymerase chain reaction (PCR) and/or serological test and/or the presence of typical symptoms such as anosmia or ageusia after exposure to a confirmed COVID-19 case, who remained asymptomatic or developed mild, self-healing, ambulatory disease.

### Next-generation sequencing

Genomic DNA was extracted from whole blood. For the 1193 patients and controls included, the whole exome (*N* = 687) or whole genome (*N* = 506) was sequenced. We used the Genome Analysis Software Kit (GATK) (version 3.4-46 or 4) best-practice pipeline to analyze our whole-exome–sequencing data (*30*). We aligned the reads obtained with the human reference genome (hg19) using the maximum exact matches algorithm in Burrows–Wheeler Aligner software (*31*). PCR duplicates were removed with Picard tools (http://broadinstitute.github.io/picard/). The GATK base quality score recalibrator was applied to correct sequencing artifacts.

All of the variants were manually curated using Integrative Genomics Viewer (IGV) and confirmed to affect the main functional protein isoform by checking the protein sequence before inclusion in further analyzes. The main functional protein isoforms were TLR3 (NM_003265), UNC93B1 (NM_030930.4), TICAM1 (NM_182919), TRAF3 (NM_145725.2), TBK1 (NM_013254.4), IRF3 (NM_001571), IRF7 (NM_001572.5), IFNAR1 (NM_000629.3), IFNAR2 (NM_001289125.3), STAT1 (NM_007315.4), STAT2 (NM_005419.4), and IRF9 (NM_006084.5). The analysis of IKBKG was customized to unmask the duplicated region in IKBKG using a specific pipeline previously described (*32*). We searched the next-generation–sequencing data for deletions in the 13 genes of interest using both the HMZDelFinder (*33*) and CANOES (*34*) algorithms.

### Statistical analysis

We performed an enrichment analysis on our cohort of 659 patients with life-threatening COVID-19 pneumonia and 534 SARS-CoV2–infected controls, focusing on 12 autosomal IFN-related genes. We considered variants that were pLOF with a MAF <0.001 (gnomAD version 2.1.1) after experimentally demonstrating that all of the pLOF variants seen in the cases were actually LOF. We compared the proportion of individuals carrying at least one pLOF variant of the 12 autosomal genes in cases and controls by means of logistic regression with the likelihood ratio test. We accounted for the ethnic heterogeneity of the cohorts by including the first three principal components of the PCA in the logistic regression model. PC adjustment is a common and efficient strategy for accounting for different ancestries of patients and controls in the study of rare variants (*35*–*38*). We checked that our adjusted burden test was well calibrated by also performing an analysis of enrichment in rare (MAF <0.001) synonymous variants of the 12 genes. PCA was performed with Plink version 1.9 software on whole-exome– and whole-genome–sequencing data and the 1000 Genomes (1kG) Project phase 3 public database as a reference, using 27,480 exonic variants with a MAF >0.01 and a call rate >0.99. The OR was also estimated by logistic regression and adjusted for ethnic heterogeneity.

### Reporter assays

Cell lines or SV40-Fib cells with known defects were transiently or stably transfected with WT, mutant variants, IFN-β- or ISRE-*firefly* luciferase reporter, and pRL-TK-*Renilla* luciferase reporter. Reporter activity was measured with the Dual-Luciferase Reporter Assay System (Promega) according to the manufacturer’s instructions. *Firefly* luciferase activity was normalized against *Renilla* luciferase activity and expressed as a fold change. TRAF3-deficient human embryonic kidney (HEK) 293T cells were kindly provided by M. Romanelli (*39*).

### pDC activation by SARS-CoV-2 and cytokine production

pDCs from an IRF7^−/−^ patient and a healthy donor matched for age and sex were cultured in the presence of medium alone, influenza virus (A/PR/8/34, 2 μg/ml; Charles River Laboratories), or the SARS-CoV-2 primary strain 220_95 (GISAID accession ID: EPI_ISL_469284) at a multiplicity of infection (MOI) of 2. After 12 hours of culture, pDC supernatant was collected for cytokine quantification. IFN-α2 levels were measured using CBA analyzis (BD Biosciences) in accordance with the manufacturer’s protocol using a 20 pg/ml detection limit. IFN-λ1 secretion was measured in an ELISA (R&D Systems, DuoSet DY7246), in accordance with the manufacturer’s instructions.

### SARS-CoV-2 infection in patient SV40-Fib

To make patient-derived fibroblasts permissive to SARS-CoV-2 infection, we delivered human ACE2 and TMPRSS2 cDNA to cells by lentivirus transduction using a modified SCRPSY vector (GenBank ID: KT368137.1). SARS-CoV-2 strain USA-WA1/2020 was obtained from BEI Resources. ACE2/TMPRSS2-transduced cells were either left untreated or treated with 500 U/ml IFN-β (11415-1, PBL Assay Science) 4 hours before infection. Cells were infected with SARS-CoV-2 (MOI = 0.5) for 1 hour at 37°C. After 24 hours of infection, cells were fixed and taken out of the BSL3 for staining.

After fixation, cells were stained with SARS-CoV-2 and ACE2 primary antibodies (0.5 and 1 μg/ml, respectively). Primary antibodies were as follows: for SARS-CoV-2, human monoclonal anti-spike-SARS-CoV-2 C121 antibody (*40*), and for ACE2, mouse monoclonal Alexa Fluor 488–conjugated antibody (FAB9332G-100UG, R&D Systems). Images were acquired with an ImageXpress Micro XLS microscope (Molecular Devices) using the 4× objective. MetaXpress software (Molecular Devices) was used to obtain single-cell mean fluorescence intensity (MFI) values.

Data analysis on single-cell MFI values was done in the R environment (version 4.0.2). ACE2/TMPRSS2-transduced cells were classified as ACE2 positive when the ACE2 log MFI was superior to the log mean MFI of mock-transduced cells plus 2.5 SDs. We excluded all wells with <150 ACE2-positive cells before SARS-CoV-2 scoring. ACE2-expressing cells were classified SARS-CoV-2 positive when the fluorescence intensity value was superior to the MFI of mock-infected cells plus 4 SDs. The median SARS-CoV-2 MFI and percentage SARS-CoV-2–positive cells were calculated for each well (independent infection).

### Single-molecule array *(*Simoa*)* IFN-α digital ELISA

Serum IFN-α concentrations were determined using Simoa technology, with reagents and procedures obtained from Quanterix Corporation (Quanterix SimoaTM IFNα Reagent Kit, Lexington, MA, USA). According to the manufacturer’s instructions, the working dilutions were 1:2 for all sera in working volumes of 170 μl.

## COVID-STORM Clinicians

Giuseppe Foti^1^, Giacomo Bellani^1^, Giuseppe Citerio^1^, Ernesto Contro^1^, Alberto Pesci^2^, Maria Grazia Valsecchi^3^, Marina Cazzaniga^4^

^1^Department of Emergency, Anesthesia and Intensive Care, School of Medicine and Surgery, University of Milano-Bicocca, San Gerardo Hospital, Monza, Italy. ^2^Department of Pneumology, School of Medicine and Surgery, University of Milano-Bicocca, San Gerardo Hospital, Monza, Italy. ^3^Center of Bioinformatics and Biostatistics, School of Medicine and Surgery, University of Milano-Bicocca, San Gerardo Hospital, Monza, Italy. ^4^Phase I Research Center, School of Medicine and Surgery, University of Milano-Bicocca, San Gerardo Hospital, Monza, Italy.

## COVID Clinicians

Jorge Abad^1^, Sergio Aguilera-Albesa^2^, Ozge Metin Akcan^3^, Ilad Alavi Darazam^4^, Juan C. Aldave^5^, Miquel Alfonso Ramos^6^, Seyed Alireza Nadji^7^, Gulsum Alkan^8^, Jerome Allardet-Servent^9^, Luis M. Allende^10^, Laia Alsina^11^, Marie-Alexandra Alyanakian^12^, Blanca Amador-Borrero^13^, Zahir Amoura^14^, Arnau Antolí^15^, Sevket Arslan^16^, Sophie Assant^17^, Terese Auguet^18^, Axelle Azot^19^, Fanny Bajolle^20^, Aurélie Baldolli^21^, Maite Ballester^22^, Hagit Baris Feldman^23^, Benoit Barrou^24^, Alexandra Beurton^25^, Agurtzane Bilbao^26^, Geraldine Blanchard-Rohner^27^, Ignacio Blanco^1^, Adeline Blandinières^28^, Daniel Blazquez-Gamero^29^, Marketa Bloomfield^30^, Mireia Bolivar-Prados^31^, Raphael Borie^32^, Cédric Bosteels^33^, Ahmed A. Bousfiha^34^, Claire Bouvattier^35^, Oksana Boyarchuk^36^, Maria Rita P. Bueno^37^, Jacinta Bustamante^20^, Juan José Cáceres Agra^38^, Semra Calimli^39^, Ruggero Capra^40^, Maria Carrabba^41^, Carlos Casasnovas^42^, Marion Caseris^43^, Martin Castelle^44^, Francesco Castelli^45^, Martín Castillo de Vera^46^, Mateus V. Castro^37^, Emilie Catherinot^47^, Martin Chalumeau^48^, Bruno Charbit^49^, Matthew P. Cheng^50^, Père Clavé^31^, Bonaventura Clotet^51^, Anna Codina^52^, Fatih Colkesen^53^, Fatma Çölkesen^54^, Roger Colobran^55^, Cloé Comarmond^56^, David Dalmau^57^, David Ross Darley^58^, Nicolas Dauby^59^, Stéphane Dauger^60^, Loic de Pontual^61^, Amin Dehban^62^, Geoffroy Delplancq^63^, Alexandre Demoule^64^, Jean-Luc Diehl^65^, Stephanie Dobbelaere^66^, Sophie Durand^67^, Waleed Eldars^68^, Mohamed Elgamal^69^, Marwa H. Elnagdy^70^, Melike Emiroglu^71^, Emine Hafize Erdeniz^72^, Selma Erol Aytekin^73^, Romain Euvrard^74^, Recep Evcen^75^, Giovanna Fabio^41^, Laurence Faivre^76^, Antonin Falck^43^, Muriel Fartoukh^77^, Morgane Faure^78^, Miguel Fernandez Arquero^79^, Carlos Flores^80^, Bruno Francois^81^, Victoria Fumadó^82^, Francesca Fusco^83^, Blanca Garcia Solis^84^, Pascale Gaussem^85^, Juana Gil-Herrera^86^, Laurent Gilardin^87^, Monica Girona Alarcon^88^, Mònica Girona-Alarcón^88^, Jean-Christophe Goffard^89^, Funda Gok^90^, Rafaela González-Montelongo^91^, Antoine Guerder^92^, Yahya Gul^93^, Sukru Nail Guner^93^, Marta Gut^94^, Jérôme Hadjadj^95^, Filomeen Haerynck^96^, Rabih Halwani^97^, Lennart Hammarström^98^, Nevin Hatipoglu^99^, Elisa Hernandez-Brito^100^, Cathérine Heijmans^101^, María Soledad Holanda-Peña^102^, Juan Pablo Horcajada^103^, Levi Hoste^104^, Eric Hoste^105^, Sami Hraiech^106^, Linda Humbert^107^, Alejandro D. Iglesias^108^, Antonio Íñigo-Campos^91^, Matthieu Jamme^109^, María Jesús Arranz^110^, Iolanda Jordan^111^, Philippe Jorens^112^, Fikret Kanat^113^, Hasan Kapakli^114^, Iskender Kara^115^, Adem Karbuz^116^, Kadriye Kart Yasar^117^, Sevgi Keles^118^, Yasemin Kendir Demirkol^119^, Adam Klocperk^120^, Zbigniew J. Król^121^, Paul Kuentz^122^, Yat Wah M. Kwan^123^, Jean-Christophe Lagier^124^, Bart N. Lambrecht^33^, Yu-Lung Lau^125^, Fleur Le Bourgeois^60^, Yee-Sin Leo^126^, Rafael Leon Lopez^127^, Daniel Leung^125^, Michael Levin^128^, Michael Levy^60^, Romain Lévy^20^, Zhi Li^49^, Agnes Linglart^129^, Bart Loeys^130^, José M. Lorenzo-Salazar^91^, Céline Louapre^131^, Catherine Lubetzki^131^, Charles-Edouard Luyt^132^, David C. Lye^133^, Davood Mansouri^134^, Majid Marjani^135^, Jesus Marquez Pereira^136^, Andrea Martin^137^, David Martínez Pueyo^138^, Javier Martinez-Picado^139^, Iciar Marzana^140^, Alexis Mathian^14^, Larissa R. B. Matos^37^, Gail V. Matthews^141^, Julien Mayaux^142^, Jean-Louis Mège^143^, Isabelle Melki^144^, Jean-François Meritet^145^, Ozge Metin^146^, Isabelle Meyts^147^, Mehdi Mezidi^148^, Isabelle Migeotte^149^, Maude Millereux^150^, Tristan Mirault^151^, Clotilde Mircher^67^, Mehdi Mirsaeidi^152^, Abián Montesdeoca Melián^153^, Antonio Morales Martinez^154^, Pierre Morange^155^, Clémence Mordacq^107^, Guillaume Morelle^156^, Stéphane Mouly^13^, Adrián Muñoz-Barrera^91^, Leslie Naesens^157^, Cyril Nafati^158^, João Farela Neves^159^, Lisa FP. Ng^160^, Yeray Novoa Medina^161^, Esmeralda Nuñez Cuadros^162^, J. Gonzalo Ocejo-Vinyals^163^, Zerrin Orbak^164^, Mehdi Oualha^20^, Tayfun Özçelik^165^, Qiang Pan-Hammarström^166^, Christophe Parizot^142^, Tiffany Pascreau^167^, Estela Paz-Artal^168^, Sandra Pellegrini^49^, Rebeca Pérez de Diego^84^, Aurélien Philippe^169^, Quentin Philippot^77^, Laura Planas-Serra^170^, Dominique Ploin^171^, Julien Poissy^172^, Géraldine Poncelet^43^, Marie Pouletty^173^, Paul Quentric^142^, Didier Raoult^143^, Anne-Sophie Rebillat^67^, Ismail Reisli^174^, Pilar Ricart^175^, Jean-Christophe Richard^176^, Nadia Rivet^28^, Jacques G. Rivière^177^, Gemma Rocamora Blanch^15^, Carlos Rodrigo^1^, Carlos Rodriguez-Gallego^178^, Agustí Rodríguez-Palmero^179^, Carolina Soledad Romero^180^, Anya Rothenbuhler^181^, Flore Rozenberg^182^, Maria Yolanda Ruiz del Prado^183^, Joan Sabater Riera^15^, Oliver Sanchez^184^, Silvia Sánchez-Ramón^185^, Agatha Schluter^170^, Matthieu Schmidt^186^, Cyril E. Schweitzer^187^, Francesco Scolari^188^, Anna Sediva^189^, Luis M. Seijo^190^, Damien Sene^13^, Sevtap Senoglu^117^, Mikko R. J. Seppänen^191^, Alex Serra Ilovich^192^, Mohammad Shahrooei^62^, Hans Slabbynck^193^, David M. Smadja^194^, Ali Sobh^195^, Xavier Solanich Moreno^15^, Jordi Solé-Violán^196^, Catherine Soler^197^, Pere Soler-Palacín^137^, Yuri Stepanovskiy^198^, Annabelle Stoclin^199^, Fabio Taccone^149^, Yacine Tandjaoui-Lambiotte^200^, Jean-Luc Taupin^201^, Simon J. Tavernier^202^, Benjamin Terrier^203^, Caroline Thumerelle^107^, Gabriele Tomasoni^204^, Julie Toubiana^48^, Josep Trenado Alvarez^205^, Sophie Trouillet-Assant^206^, Jesús Troya^207^, Alessandra Tucci^208^, Matilde Valeria Ursini‬^83^, Yurdagul Uzunhan^209^, Pierre Vabres^210^, Juan Valencia-Ramos^211^, Eva Van Braeckel^33^, Stijn Van de Velde^212^, Ana Maria Van Den Rym^84^, Jens Van Praet^213^, Isabelle Vandernoot^214^, Hulya Vatansev^215^, Valentina Vélez-Santamaria^42^, Sébastien Viel^171^, Cédric Vilain^216^, Marie E. Vilaire^67^, Audrey Vincent^35^, Guillaume Voiriot^217^, Fanny Vuotto^107^, Alper Yosunkaya^90^, Barnaby E. Young^126^, Fatih Yucel^218^, Faiez Zannad^219^, Mayana Zatz^37^, Alexandre Belot^220^*

^1^University Hospital and Research Institute “Germans Trias i Pujol,” Badalona, Spain. ^2^Navarra Health Service Hospital, Pamplona, Spain. ^3^Division of Pediatric Infectious Diseases, Necmettin Erbakan University, Meram Medical Faculty, Konya, Turkey. ^4^Department of Infectious Diseases, Loghman Hakim Hospital, Shahid Beheshti University of Medical Sciences, Tehran, Iran. ^5^Hospital Nacional Edgardo Rebagliati Martins, Lima, Peru. ^6^Parc Sanitari Sant Joan de Déu, Sant Boi de Llobregat, Spain. ^7^Virology Research Center, National Institutes of Tuberculosis and Lung Diseases, Shahid Beheshti University of Medical Sciences, Tehran, Iran. ^8^Division of Pediatric Infectious Diseases, Faculty of Medicine, Selcuk University, Konya, Turkey. ^9^Intensive Care Unit, Hôpital Européen, Marseille, France. ^10^Immunology Department, University Hospital 12 de Octubre, Research Institute imas12, and Complutense University, Madrid, Spain. ^11^Clinical Immuology and Primary Immunodeficiencies Unit, Hospital Sant Joan de Déu, Barcelona, Spain. ^12^Department of Biological Immunology, Necker Hospital for Sick Children, APHP and INEM, Paris, France. ^13^Internal Medicine Department, Hôpital Lariboisière, APHP; Université de Paris, Paris, France. ^14^Internal Medicine Department, Pitié-Salpétrière Hospital, Paris, France. ^15^Hospital Universitari de Bellvitge, Barcelona, Spain. ^16^Division of Clinical Immunology and Allergy, Necmettin Erbakan University, Meram Medical Faculty, Konya, Turkey. ^17^Joint Research Unit, Hospices Civils de Lyon-bio Mérieux, Hospices Civils de Lyon, Lyon Sud Hospital, Lyon, France. ^18^Hospital U. de Tarragona Joan XXIII, Universitat Rovira i Virgili (URV), IISPV, Tarragona, Spain. ^19^Private practice, Paris, France. ^20^Necker Hospital for Sick Children, AP-HP, Paris, France. ^21^Department of Infectious Diseases, CHU de Caen, Caen, France. ^22^Consorcio Hospital General Universitario, Valencia, Spain. ^23^The Genetics Institute, Tel Aviv Sourasky Medical Center and Sackler Faculty of Medicine, Tel Aviv University, Tel Aviv, Israel. ^24^Department of Urology, Nephrology, and Transplantation, APHP-SU, Sorbonne Université, INSERM U 1082, Paris, France. ^25^Service de Médecine Intensive–Réanimation et Pneumologie, APHP Hôpital Pitié–Salpêtrière, Paris, France. ^26^Cruces University Hospital, Bizkaia, Spain. ^27^Paediatric Immunology and Vaccinology Unit, Geneva University Hospitals and Faculty of Medicine, Geneva, Switzerland. ^28^Hematology, Georges Pompidou Hospital, APHP, Paris, France. ^29^Pediatric Infectious Diseases Unit, Instituto de Investigación 12 de Octubre imas12, and Hospital Universitario 12 de Octubre, Madrid, Spain. ^30^Department of Immunology, Motol University Hospital, 2nd Faculty of Medicine, Charles University, Department of Pediatrics, Thomayer’s Hospital, 1st Faculty of Medicine, Charles University, Prague, Czech Republic. ^31^Centro de Investigación Biomédica en Red de Enfermedades Hepàticas y Digestivas (Ciberehd), Hospital de Mataró, Consorci Sanitari del Maresme, Mataró, Spain. ^32^Service de Pneumologie, Hopital Bichat, APHP, Paris, France. ^33^Department of Pulmonology, Ghent University Hospital, Ghent, Belgium. ^34^Clinical Immunology Unit, Pediatric Infectious Disease Department, Faculty of Medicine and Pharmacy, Averroes University Hospital, LICIA Laboratoire d’Immunologie Clinique, d’Inflammation et d’Allergie, Hassann Ii University, Casablanca, Morocco. ^35^Endocrinology Unit, APHP Hôpitaux Universitaires Paris-Sud, Le Kremlin-Bicêtre, France. ^36^Department of Children’s Diseases and Pediatric Surgery, I. Horbachevsky Ternopil National Medical University, Ternopil, Ukraine. ^37^Human Genome and Stem-Cell Research Center, University of São Paulo, São Paulo, Brazil. ^38^Hospital Insular, Las Palmas de Gran Canaria, Spain. ^39^Division of Critical Care Medicine, Department of Anesthesiology and Reanimation, Konya State Hospital, Konya, Turkey. ^40^MS Center, Spedali Civili, Brescia, Italy. ^41^Fondazione IRCCS Ca’ Granda Ospedale Maggiore Policlinico, Milan, Italy. ^42^Bellvitge University Hospital, L’Hospitalet de Llobregat, Barcelona, Spain. ^43^Hopital Robert Debré, Paris, France. ^44^Pediatric Immuno-hematology Unit, Necker Enfants Malades Hospital, AP-HP, Paris, France. ^45^Department of Infectious and Tropical Diseases, University of Brescia, ASST Spedali Civili di Brescia, Brescia, Italy. ^46^Doctoral Health Care Center, Canarian Health System, Las Palmas de Gran Canaria, Spain. ^47^Hôpital Foch, Suresnes, France. ^48^Necker Hospital for Sick Children, Paris University, AP-HP, Paris, France. ^49^Pasteur Institute, Paris, France. ^50^McGill University Health Centre, Montreal, Canada. ^51^University Hospital and Research Institute “Germans Trias i Pujol,” IrsiCaixa AIDS Research Institute, UVic-UCC, Badalona, Spain. ^52^Clinical Biochemistry, Pathology, Paediatric Neurology and Molecular Medicine Departments and Biobank, Institut de Recerca Sant Joan de Déu and CIBERER-ISCIII, Esplugues, Spain. ^53^Division of Clinical Immunology and Allergy, Department of Internal Medicine, Necmettin Erbakan University, Meram Medical Faculty, Konya, Turkey. ^54^Department of Infectious Diseases and Clinical Microbiology, Konya Training and Research Hospital, Konya, Turkey. ^55^Hospital Universitari Vall d’Hebron, Barcelona, Spain. ^56^Pitié-Salpêtrière Hospital, Paris, France. ^57^Fundació Docència i Recerca Mútua Terrassa, Barcelona, Spain; Hospital Universitari Mutua Terrassa, Universitat de Barcelona, Terrassa, Catalonia, Spain. ^58^UNSW Medicine, St. Vincent’s Clinical School, and Department of Thoracic Medicine, St. Vincent’s Hospital Darlinghurst, Sidney, Australia. ^59^CHU Saint-Pierre, Université Libre de Bruxelles, Brussels, Belgium. ^60^Pediatric Intensive Care Unit, Robert-Debré University Hospital, APHP, Paris, France. ^61^Sorbonne Paris Nord, Hôpital Jean Verdier, APHP, Bondy, France. ^62^Specialized Immunology Laboratory of Dr. Shahrooei, Sina Medical Complex, Ahvaz, Iran. ^63^Centre de Génétique Humaine, CHU Besançon, Besançon, France. ^64^Sorbonne Université Médecine and APHP Sorbonne Université Site Pitié-Salpêtrière, Paris, France. ^65^Intensive Care Unit, Georges Pompidou Hospital, APHP, Paris, France. ^66^Department of Pneumology, AZ Delta, Roeselare, Belgium. ^67^Institut Jérôme Lejeune, Paris, France. ^68^Department of Microbiology and Immunology, Faculty of Medicine, Mansoura University, Mansoura, Egypt. ^69^Department of Chest, Faculty of Medicine, Mansoura University, Mansoura, Egypt. ^70^Department of Medical Biochemistry and Molecular Biology, Faculty of Medicine, Mansoura University, Mansoura, Egypt. ^71^Faculty of Medicine, Division of Pediatric Infectious Diseases, Selcuk University, Konya, Turkey. ^72^Division of Pediatric Infectious Diseases, Ondokuz Mayıs University, Samsun, Turkey. ^73^Necmettin Erbakan University, Meram Medical Faculty, Division of Pediatric Allergy and Immunology, Konya, Turkey. ^74^Centre Hospitalier Fleyriat, Bourg-en-Bresse, France. ^75^Division of Clinical Immunology and Allergy, Department of Internal Medicine, Necmettin Erbakan University, Meram Medical Faculty, Konya, Turkey. ^76^Centre de Génétique, CHU Dijon, Dijon, France. ^77^APHP Tenon Hospital, Paris, France. ^78^Sorbonne Universités, UPMC University of Paris, Paris, France. ^79^Department of Clinical Immunology , Hospital Clínico San Carlos, Madrid, Spain. ^80^Genomics Division, Instituto Tecnológico y de Energías Renovables (ITER), Santa Cruz de Tenerife, Spain; CIBER de Enfermedades Respiratorias, Instituto de Salud Carlos III, Madrid, Spain; Research Unit, Hospital Universitario N.S. de Candelaria, Santa Cruz de Tenerife, Spain; Instituto de Tecnologías Biomédicas (ITB), Universidad de La Laguna, San Cristóbal de La Laguna, Spain. ^81^CHU Limoges and Inserm CIC 1435 and UMR 1092, Limoges, France. ^82^Infectious Diseases Unit, Department of Pediatrics, Hospital Sant Joan de Déu, Barcelona, Spain; Institut de Recerca Sant Joan de Déu, Spain; Universitat de Barcelona (UB), Barcelona, Spain. ^83^Institute of Genetics and Biophysics “Adriano Buzzati-Traverso,” IGB-CNR, Naples, Italy. ^84^Laboratory of Immunogenetics of Human Diseases, IdiPAZ Institute for Health Research, La Paz Hospital, Madrid, Spain. ^85^Hematology, APHP, Hopital Européen Georges Pompidou and Inserm UMR-S1140, Paris, France. ^86^Hospital General Universitario and Instituto de Investigación Sanitaria “Gregorio Marañón,” Madrid, Spain. ^87^Bégin military Hospital, Bégin, France. ^88^Pediatric Intensive Care Unit, Hospital Sant Joan de Déu, Barcelona, Spain. ^89^Department of Internal Medicine, Hôpital Erasme, Université Libre de Bruxelles, Brussels, Belgium. ^90^Division of Critical Care Medicine, Department of Anesthesiology and Reanimation, Necmettin Erbakan University, Meram Medical Faculty, Konya, Turkey. ^91^Genomics Division, Instituto Tecnológico y de Energías Renovables (ITER), Santa Cruz de Tenerife, Spain. ^92^Assistance Publique Hôpitaux de Paris, Paris, France. ^93^Division of Allergy and Immunology, Necmettin Erbakan University, Meram Medical Faculty, Konya, Turkey. ^94^CNAG-CRG, Centre for Genomic Regulation (CRG), Barcelona Institute of Science and Technology (BIST); Universitat Pompeu Fabra (UPF), Barcelona, Spain. ^95^Department of Internal Medicine, National Reference Center for Rare Systemic Autoimmune Diseases, AP-HP, APHP-CUP, Hôpital Cochin, Paris, France. ^96^Ghent University Hospital, Ghent, Belgium. ^97^Sharjah Institute of Medical Research, College of Medicine, University of Sharjah, Sharjah, UAE. ^98^Department of Laboratory Medicine, SE14186, Huddinge, Karolinska Institutet, Stockholm, Sweden. ^99^Pediatric Infectious Diseases Unit, Bakirkoy Dr. Sadi Konuk Training and Research Hospital, University of Health Sciences, Istanbul, Turkey. ^100^Department of Immunology, Hospital Universitario de Gran Canaria Dr. Negrín, Canarian Health System, Las Palmas de Gran Canaria, Spain. ^101^Department of Pediatric Hemato-Oncology, Jolimont Hospital; Department of Pediatric Hemato-Oncology, HUDERF, Brussels, Belgium. ^102^Intensive Care Unit, Marqués de Valdecilla Hospital, Santander, Spain. ^103^Hospital del Mar, Parc de Salut Mar, Barcelona, Spain. ^104^Department of Pediatric Pulmonology and Immunology, Ghent University Hospital, Ghent, Belgium. ^105^Department of Intensive Care Unit, Ghent University Hospital, Ghent, Belgium. ^106^Intensive Care Unit, APHM, Marseille, France. ^107^CHU Lille, Lille, France. ^108^Department of Pediatrics, Columbia University, New York, NY, USA. ^109^Centre Hospitalier Intercommunal Poissy Saint Germain en Laye, Poissy, France. ^110^Fundació Docència i Recerca Mútua Terrassa, Terrassa, Spain. ^111^Hospital Sant Joan de Déu, Kids Corona Platfform, Barcelona, Spain. ^112^Department of Intensive Care Unit, University Hospital Antwerp, Antwerp, Belgium. ^113^Selcuk University, Faculty of Medicine, Chest Diseases Department, Konya, Turkey. ^114^Division of Allergy and Immunology, Balikesir Ataturk City Hospital, Balikesir, Turkey. ^115^Division of Critical Care Medicine, Selcuk University, Faculty of Medicine, Konya, Turkey. ^116^Division of Pediatric Infectious Diseases, Prof. Dr. Cemil Tascıoglu City Hospital, Istanbul, Turkey. ^117^Departments of Infectious Diseases and Clinical Microbiology, Bakirkoy Dr. Sadi Konuk Training and Research Hospital, University of Health Sciences, Istanbul, Turkey. ^118^Meram Medical Faculty, Necmettin Erbakan University, Meram Medical Faculty, Konya, Turkey. ^119^Health Sciences University, Umraniye Education and Research Hospital, Istanbul, Turkey. ^120^Department of Immunology, 2nd Faculty of Medicine, Charles University and University Hospital in Motol, Prague, Czech Republic. ^121^Central Clinical Hospital of Ministry of the Interior and Administration in Warsaw, Warsaw, Poland. ^122^Oncobiologie Génétique Bioinformatique, PC Bio, CHU Besançon, Besançon, France. ^123^Paediatric Infectious Disease Unit, Hospital Authority Infectious Disease Center, Princess Margaret Hospital, Hong Kong (Special Administrative Region), China. ^124^Aix Marseille University, IRD, MEPHI, IHU Méditerranée Infection, Marseille, France. ^125^Department of Paediatrics and Adolescent Medicine, The University of Hong Kong, Hong Kong, China. ^126^National Centre for Infectious Diseases, Singapore. ^127^Hospital Universitario Reina Sofía, Cordoba, Spain. ^128^Imperial College, London, UK. ^129^Endocrinology and Diabetes for Children, AP-HP, Bicêtre Paris-Saclay Hospital, Le Kremlin-Bicêtre, France. ^130^Department of Medical Genetics, University Hospital Antwerp, Antwerp, Belgium. ^131^Neurology Unit, APHP Pitié-Salpêtrière Hospital, Paris University, Paris, France. ^132^Intensive Care Unit, APHP Pitié-Salpêtrière Hospital, Paris University, Paris, France. ^133^National Centre for Infectious Diseases; Tan Tock Seng Hospital; Yong Loo Lin School of Medicine; Lee Kong Chian School of Medicine, Singapore. ^134^Department of Clinical Immunology and Infectious Diseases, National Research Institute of Tuberculosis and Lung Diseases, Shahid Beheshti University of Medical Sciences, Tehran, Iran. ^135^Clinical Tuberculosis and Epidemiology Research Center, National Research Institute of Tuberculosis and Lung Diseases (NRITLD), Shahid Beheshti University of Medical Sciences, Tehran, Iran. ^136^Hospital Sant Joan de Déu and University of Barcelona, Barcelona, Spain. ^137^Pediatric Infectious Diseases and Immunodeficiencies Unit, Hospital Universitari Vall d’Hebron, Vall d’Hebron Research Institute, Vall d’Hebron Barcelona Hospital Campus. Universitat Autònoma de Barcelona (UAB), Barcelona, Spain. ^138^Hospital Universitari Mutua de Terrassa, Universitat de Barcelona, Barcelona, Spain. ^139^IrsiCaixa AIDS Research Institute, ICREA, UVic-UCC, Research Institute “Germans Trias i Pujol,” Badalona, Spain. ^140^Department of Laboratory, Cruces University Hospital, Barakaldo, Bizkaia, Spain. ^141^University of New South Wales, New South Wales, Australia. ^142^APHP Pitié-Salpêtrière Hospital, Paris, France. ^143^Aix-Marseille University, APHM, Marseille, France. ^144^Robert Debré Hospital, Paris, France. ^145^APHP Cohin Hospital, Paris, France. ^146^Necmettin Erbakan University Meram Faculty of Medicine Department of Pediatric Infectious Diseases, Konya, Turkey. ^147^University Hospitals Leuven, Leuven, Belgium. ^148^Hospices Civils de Lyon, Hôpital de la Croix-Rousse, Lyon, France. ^149^Hôpital Erasme, Brussels, Belgium. ^150^CH Gonesse, Gonesse, France. ^151^Vascular Medicine, Georges Pompidou Hospital, APHP, Paris, France. ^152^Division of Pulmonary and Critical Care, University of Miami, Miami, FL, USA. ^153^Guanarteme Health Care Center, Canarian Health System, Las Palmas de Gran Canaria, Spain. ^154^Regional University Hospital of Malaga, Malaga, Spain. ^155^Aix-Marseille Université, Marseille, France. ^156^Department of General Paediatrics, Hôpital Bicêtre, AP-HP, University of Paris Saclay, Le Kremlin-Bicêtre, France. ^157^Department of Internal Medicine, Ghent University Hospital, Ghent, Belgium. ^158^CHU de La Timone, Marseille, France. ^159^Centro Hospitalar Universitário de Lisboa Central, Lisbon, Portugal. ^160^Infectious Diseases Horizontal Technlogy Centre, A*STAR; Singapore Immunology Network, A*STAR, Singapore. ^161^Department of Pediatrics, Complejo Hospitalario Universitario Insular-Materno Infantil, Canarian Health System, Las Palmas de Gran Canaria, Spain. ^162^Regional Universitary Hospital of Málaga, Málaga, Spain. ^163^Hospital Universitario Marqués de Valdecilla, Santander, Spain. ^164^Faculty of Medicine, Ataturk University, Erzurum, Turkey. ^165^Department of Molecular Biology and Genetics, Bilkent University, Ankara, Turkey. ^166^Department of Biosciences and Nutrition, Karolinska Institutet, SE14183, Stockholm, Sweden. ^167^L’Hôpital Foch, Suresnes, France. ^168^Department of Immunology, Hospital Universitario 12 de Octubre, Instituto de Investigación Sanitaria Hospital 12 de Octubre imas12, Madrid, Spain. ^169^APHP Hôpitaux Universitaires Paris-Sud, Le Kremlin-Bicêtre, France. ^170^Neurometabolic Diseases Laboratory, IDIBELL-Hospital Duran i Reynals, Barcelona; CIBERER U759, ISCiii, Madrid, Spain. ^171^Hospices Civils de Lyon, Lyon, France. ^172^Université de Lille, Inserm U1285, CHU Lille, Paris, France. ^173^Departement of General Pediatrics, University Hospital Robert Debré, APHP, Paris, France. ^174^Necmettin Erbakan University, Konya, Turkey. ^175^Germans Trias i Pujol Hospital, Badalona, Spain. ^176^Medical Intensive Care Unit, Hopital de la Croix-Rousse, Hospices Civils de Lyon, Lyon, France. ^177^Pediatric Infectious Diseases and Immunodeficiencies Unit, Hospital Universitari Vall d’Hebron, Vall d’Hebron Research Institute, Vall d’Hebron Barcelona Hospital Campus., Barcelona, Spain. ^178^Department of Immunology, Hospital Universitario de Gran Canaria Dr. Negrín, Canarian Health System, Las Palmas de Gran Canaria, Spain. University Fernando Pessoa Canarias, Las Palmas de Gran Canaria, Spain. ^179^Neurometabolic Diseases Laboratory, IDIBELL-Hospital Duran i Reynals, Barcelona, Spain. ^180^Consorcio Hospital General Universitario, Valencia, Spain. ^181^APHP Hôpitaux Universitaires Paris-Sud, Paris, France. ^182^Virology Unit, Université de Paris, Cohin Hospital, APHP, Paris, France. ^183^Hospital San Pedro, Logroño, Spain. ^184^Respiratory Medicine, Georges Pompidou Hospital, APHP, Paris, France. ^185^Department of Immunology, Hospital Clínico San Carlos, Madrid, Spain. ^186^Service de Médecine Intensive Réanimation, Institut de Cardiologie, Hopital Pitié-Salpêtrière, Paris, France. ^187^CHRU de Nancy, Hôpital d’Enfants, Vandoeuvre, France. ^188^Chair of Nephrology, University of Brescia, Brescia, Italy. ^189^Department of Immunology, 2nd Faculty of Medicine, Charles University and Motol University Hospital, Prague, Czech Republic. ^190^Clínica Universidad de Navarra, Madrid, Spain. ^191^HUS Helsinki University Hospital, Children and Adolescents, Rare Disease Center, and Inflammation Center, Adult Immunodeficiency Unit, Majakka, Helsinki, Finland. ^192^Fundació Docència i Recerca Mútua Terrassa, Terrassa, Spain. ^193^Department of Pulmonology, ZNA Middelheim, Antwerp, Belgium. ^194^INSERM UMR-S 1140, Biosurgical Research Lab (Carpentier Foundation), Paris University and Hopital Européen Georges Pompidou, Paris, France. ^195^Department of Pediatrics, Faculty of Medicine, Mansoura University, Mansoura, Egypt. ^196^Critical Care Unit, Hospital Universitario de Gran Canaria Dr. Negrín, Canarian Health System, Las Palmas de Gran Canaria, Spain. ^197^CHU de Saint Etienne, Saint-Priest-en-Jarez, France. ^198^Shupyk National Medical Academy for Postgraduate Education, Kiev, Ukraine. ^199^Gustave Roussy Cancer Campus, Villejuif, France. ^200^Intensive Care Unit, Avicenne Hospital, APHP, Bobigny, France. ^201^Laboratory of Immunology and Histocompatibility, Saint-Louis Hospital, Paris University, Paris, France. ^202^Department of Internal Diseases and Pediatrics, Primary Immune Deficiency Research Lab, Centre for Primary Immunodeficiency Ghent, Jeffrey Modell Diagnosis and Research Centre, Ghent University Hospital, Ghent, Belgium. ^203^Department of Internal Medicine, Université de Paris, INSERM, U970, PARCC, F-75015, Paris, France. ^204^First Division of Anesthesiology and Critical Care Medicine, University of Brescia, ASST Spedali Civili di Brescia, Brescia, Italy. ^205^Intensive Care Department, Hospital Universitari Mutua Terrassa, Universitat Barcelona, Terrassa, Spain. ^206^Hospices Civils de Lyon, Lyon Sud Hospital, Lyon, France. ^207^Infanta Leonor University Hospital, Madrid, Spain. ^208^Hematology Department, ASST Spedali Civili di Brescia, Brescia, Italy. ^209^Pneumologie, Hôpital Avicenne, APHP, INSERM U1272, Université Sorbonne Paris Nord, Bobigny, France. ^210^Dermatology Unit, Laboratoire GAD, INSERM UMR1231 LNC, Université de Bourgogne, Dijon, France. ^211^University Hospital of Burgos, Burgos, Spain. ^212^Intensive Care Unit, M. Middelares Ghent, Ghent, Belgium. ^213^Department of Nephrology and Infectiology, AZ Sint-Jan Brugge-Oostende AV, Bruges, Belgium. ^214^Center of Human Genetics, Hôpital Erasme, Université Libre de Bruxelles, Brussels, Belgium. ^215^Department of Chest Diseases, Necmettin Erbakan University, Meram Medical Faculty, Konya, Turkey. ^216^CHU de Caen, Caen, France. ^217^Sorbonne Université, Service de Médecine Intensive Réanimation, Hôpital Tenon, Assistance Publique-Hôpitaux de Paris, Paris, France. ^218^General Intensive Care Unit, Konya Training and Research Hospital, Konya, Turkey. ^219^CHU de Nancy, Nancy, France. ^220^University of Lyon, CIRI, INSERM U1111, National Referee Centre RAISE, Pediatric Rheumatology, HFME, Hospices Civils de Lyon, Lyon, France.

*Leader of COVID Clinicians.

## Imagine COVID Group

Christine Bole-Feysot, Stanislas Lyonnet*, Cécile Masson, Patrick Nitschke, Aurore Pouliet, Yoann Schmitt, Frederic Tores, Mohammed Zarhrate

Imagine Institute, Université de Paris, INSERM UMR 1163, Paris, France.

*Leader of the Imagine COVID Group.

## French COVID Cohort Study Group

Laurent Abel^1^, Claire Andrejak^2^, François Angoulvant^3^, Delphine Bachelet^4^, Romain Basmaci^5^, Sylvie Behillil^6^, Marine Beluze^7^, Dehbia Benkerrou^8^, Krishna Bhavsar^4^, François Bompart^9^, Lila Bouadma^4^, Maude Bouscambert^10^, Mireille Caralp^11^, Minerva Cervantes-Gonzalez^12^, Anissa Chair^4^, Alexandra Coelho^13^, Camille Couffignal^4^, Sandrine Couffin-Cadiergues^14^, Eric D’Ortenzio^12^, Charlene Da Silveira^4^, Marie-Pierre Debray^4^, Dominique Deplanque^15^, Diane Descamps^16^, Mathilde Desvallées^17^, Alpha Diallo^18^, Alphonsine Diouf^13^, Céline Dorival^8^, François Dubos^19^, Xavier Duval^4^, Philippine Eloy^4^, Vincent VE Enouf^20^, Hélène Esperou^21^, Marina Esposito-Farese^4^, Manuel Etienne^22^, Nadia Ettalhaoui^4^, Nathalie Gault^4^, Alexandre Gaymard^10^, Jade Ghosn^4^, Tristan Gigante^23^, Isabelle Gorenne^4^, Jérémie Guedj^24^, Alexandre Hoctin^13^, Isabelle Hoffmann^4^, Salma Jaafoura^21^, Ouifiya Kafif^4^, Florentia Kaguelidou^25^, Sabina Kali^4^, Antoine Khalil^4^, Coralie Khan^17^, Cédric Laouénan^4^, Samira Laribi^4^, Minh Le^4^, Quentin Le Hingrat^4^, Soizic Le Mestre^18^, Hervé Le Nagard^24^, François-Xavier Lescure^4^, Yves Lévy^26^, Claire Levy-Marchal^27^, Bruno Lina^10^, Guillaume Lingas^24^, Jean Christophe Lucet^4^, Denis Malvy^28^, Marina Mambert^13^, France Mentré^4^, Noémie Mercier^18^, Amina Meziane^8^, Hugo Mouquet^20^, Jimmy Mullaert^4^, Nadège Neant^24^, Marion Noret^29^, Justine Pages^30^, Aurélie Papadopoulos^21^, Christelle Paul^18^, Nathan Peiffer-Smadja^4^, Ventzislava Petrov-Sanchez^18^, Gilles Peytavin^4^, Olivier Picone^31^, Oriane Puéchal^12^, Manuel Rosa-Calatrava^10^, Bénédicte Rossignol^23^, Patrick Rossignol^32^, Carine Roy^4^, Marion Schneider^4^, Caroline Semaille^12^, Nassima Si Mohammed^4^, Lysa Tagherset^4^, Coralie Tardivon^4^, Marie-Capucine Tellier^4^, François Téoulé^8^, Olivier Terrier^10^, Jean-François Timsit^4^, Théo Trioux^4^, Christelle Tual^33^, Sarah Tubiana^4^, Sylvie van der Werf^34^, Noémie Vanel^35^, Aurélie Veislinger^33^, Benoit Visseaux^16^, Aurélie Wiedemann^26^, Yazdan Yazdanpanah^36^

^1^Inserm UMR 1163, Paris, France. ^2^CHU Amiens, France. ^3^Hôpital Necker, Paris, France. ^4^Hôpital Bichat, Paris, France. ^5^Hôpital Louis Mourrier, Colombes, France. ^6^Institut Pasteur, Paris, France. ^7^F-CRIN Partners Platform, AP-HP, Université de Paris, Paris, France. ^8^Inserm UMR 1136, Paris, France. ^9^Drugs for Neglected Diseases Initiative, Geneva, Switzerland. ^10^Inserm UMR 1111, Lyon, France. ^11^Inserm Transfert, Paris, France. ^12^REACTing, Paris, France. ^13^Inserm UMR 1018, Paris, France. ^14^Inserm, Pôle Recherche Clinique, Paris, France. ^15^CIC 1403 Inserm-CHU Lille, Paris, France. ^16^Université de Paris, IAME, INSERM UMR 1137, AP-HP, University Hospital Bichat Claude Bernard, Virology, Paris, France. ^17^Inserm UMR 1219, Bordeaux, France. ^18^ANRS, Paris, France. ^19^CHU Lille, Lille, France. ^20^Pasteur Institute, Paris, France. ^21^Inserm sponsor, Paris, France. ^22^CHU Rouen–SMIT, Rouen, France. ^23^FCRIN INI-CRCT, Nancy, France. ^24^Inserm UMR 1137, Paris, France. ^25^Centre d’Investigation Clinique, Inserm CIC1426, Hôpital Robert Debré, Paris, France. ^26^Inserm UMR 955, Créteil, France; Vaccine Research Instiute (VRI), Paris, France. ^27^F-CRIN INI-CRCT, Paris, France. ^28^CHU de Bordeaux–SMIT, Bordeaux, France. ^29^RENARCI, Annecy, France. ^30^Hôpital Robert Debré, Paris, France. ^31^Hôpital Louis Mourier–Gynécologie, Colombes, France. ^32^University of Lorraine, Plurithematic Clinical Investigation Centre Inserm CIC-P; 1433, Inserm U1116, CHRU Nancy Hopitaux de Brabois, F-CRIN INI-CRCT (Cardiovascular and Renal Clinical Trialists), Nancy, France. ^33^Inserm CIC-1414, Rennes, France. ^34^Institut Pasteur, UMR 3569 CNRS, Université de Paris, Paris, France. ^35^Hôpital la Timone, Marseille, France. ^36^Bichat–SMIT, Paris, France.

## CoV-Contact Cohort

Loubna Alavoine^1^, Karine K. A. Amat^2^, Sylvie Behillil^3^, Julia Bielicki^4^, Patricia Bruijning^5^, Charles Burdet^6^, Eric Caumes^7^, Charlotte Charpentier^8^, Bruno Coignard^9^, Yolande Costa^1^, Sandrine Couffin-Cadiergues^10^, Florence Damond^8^, Aline Dechanet^11^, Christelle Delmas^10^, Diane Descamps^8^, Xavier Duval^1^, Jean-Luc Ecobichon^1^, Vincent Enouf^3^, Hélène Espérou^10^, Wahiba Frezouls^1^, Nadhira Houhou^11^, Emila Ilic-Habensus^1^, Ouifiya Kafif^11^, John Kikoine^11^, Quentin Le Hingrat^8^, David Lebeaux^12^, Anne Leclercq^1^, Jonathan Lehacaut^1^, Sophie Letrou^1^, Bruno Lina^13^, Jean-Christophe Lucet^14^, Denis Malvy^15^, Pauline Manchon^11^, Milica Mandic^1^, Mohamed Meghadecha^16^, Justina Motiejunaite^17^, Mariama Nouroudine^1^, Valentine Piquard^11^, Andreea Postolache^11^, Caroline Quintin^1^, Jade Rexach^1^, Layidé Roufai^10^, Zaven Terzian^11^, Michael Thy^18^, Sarah Tubiana^1^, Sylvie van der Werf^3^, Valérie Vignali^1^, Benoit Visseaux^8^, Yazdan Yazdanpanah^14^

^1^Centre d’Investigation Clinique, Inserm CIC 1425, Hôpital Bichat Claude Bernard, APHP, Paris, France. ^2^IMEA Fondation Léon M’Ba, Paris, France. ^3^Institut Pasteur, UMR 3569 CNRS, Université de Paris, Paris, France. ^4^University of Basel Children’s Hospital. ^5^Julius Center for Health Sciences and Primary Care, Utrecht, Netherlands. ^6^Université de Paris, IAME, Inserm UMR 1137, F-75018, Paris, France, Hôpital Bichat Claude Bernard, APHP, Paris, France. ^7^Hôpital Pitiè Salpétriere, APHP, Paris. ^8^Université de Paris, IAME, INSERM UMR 1137, AP-HP, University Hospital Bichat Claude Bernard, Virology, Paris, France. ^9^Santé Publique France, Saint Maurice, France. ^10^Pole Recherche Clinique, Inserm, Paris, France. ^11^Hôpital Bichat Claude Bernard, APHP, Paris, France. ^12^APHP, Paris, France. ^13^Virpath Laboratory, International Center of Research in Infectiology, Lyon University, INSERM U1111, CNRS UMR 5308, ENS, UCBL, Lyon, France. ^14^IAME Inserm UMR 1138, Hôpital Bichat Claude Bernard, APHP, Paris, France. ^15^Service des Maladies Infectieuses et Tropicales; Groupe Pellegrin-Place Amélie-Raba-Léon, Bordeaux, France. ^16^Hôpital Hotel Dieu, APHP, Paris, France. ^17^Service des Explorations Fonctionnelles, Hôpital Bichat–Claude Bernard, APHP, Paris, France. ^18^Center for Clinical Investigation, Assistance Publique-Hôpitaux de Paris, Bichat-Claude Bernard University Hospital, Paris, France.

## Amsterdam UMC Covid-19 Biobank

Michiel van Agtmael^1^, Anna Geke Algera^2^, Frank van Baarle^2^, Diane Bax^3^, Martijn Beudel^4^, Harm Jan Bogaard^5^, Marije Bomers^1^, Lieuwe Bos^2^, Michela Botta^2^, Justin de Brabander^6^, Godelieve de Bree^6^, Matthijs C. Brouwer^4^, Sanne de Bruin^2^, Marianna Bugiani^7^, Esther Bulle^2^, Osoul Chouchane^1^, Alex Cloherty^3^, Paul Elbers^2^, Lucas Fleuren^2^, Suzanne Geerlings^1^, Bart Geerts^8^, Theo Geijtenbeek^9^, Armand Girbes^2^, Bram Goorhuis^1^, Martin P. Grobusch^1^, Florianne Hafkamp^9^, Laura Hagens^2^, Jorg Hamann^10^, Vanessa Harris^1^, Robert Hemke^11^, Sabine M. Hermans^1^, Leo Heunks^2^, Markus W. Hollmann^8^, Janneke Horn^2^, Joppe W. Hovius^1^, Menno D. de Jong^12^, Rutger Koning^4^, Niels van Mourik^2^, Jeaninne Nellen^1^, Frederique Paulus^2^, Edgar Peters^1^, Tom van der Poll^1^, Benedikt Preckel^8^, Jan M. Prins^1^, Jorinde Raasveld^2^, Tom Reijnders^1^, Michiel Schinkel^1^, Marcus J. Schultz^2^, Alex Schuurman^13^, Kim Sigaloff^1^, Marry Smit^2^, Cornelis S. Stijnis^1^, Willemke Stilma^2^, Charlotte Teunissen^14^, Patrick Thoral^2^, Anissa Tsonas^2^, Marc van der Valk^1^, Denise Veelo^8^, Alexander P.J. Vlaar^15^, Heder de Vries^2^, Michèle van Vugt^1^, W. Joost Wiersinga^1^, Dorien Wouters^16^, A. H. (Koos) Zwinderman^17^, Diederik van de Beek^4^*

^1^Department of Infectious Diseases, Amsterdam UMC, Amsterdam, Netherlands. ^2^Department of Intensive Care, Amsterdam UMC, Amsterdam, Netherlands. ^3^Experimental Immunology, Amsterdam UMC, Amsterdam, Netherlands. ^4^Department of Neurology, Amsterdam UMC, Amsterdam Neuroscience, Amsterdam, Netherlands. ^5^Department of Pulmonology, Amsterdam UMC, Amsterdam, Netherlands. ^6^Department of Infectious Diseases, Amsterdam UMC, Amsterdam, Netherlands. ^7^Department of Pathology, Amsterdam UMC, Amsterdam, Netherlands. ^8^Department of Anesthesiology, Amsterdam UMC, Amsterdam, Netherlands. ^9^Department of Experimental Immunology, Amsterdam UMC, Amsterdam, Netherlands. ^10^Amsterdam UMC Biobank Core Facility, Amsterdam UMC, Amsterdam, Netherlands. ^11^Department of Radiology, Amsterdam UMC, Amsterdam, Netherlands. ^12^Department of Medical Microbiology, Amsterdam UMC, Amsterdam, Netherlands. ^13^Department of Internal Medicine, Amsterdam UMC, Amsterdam, Netherlands. ^14^Neurochemical Laboratory, Amsterdam UMC, Amsterdam, Netherlands. ^15^Department of Intensive Care, Amsterdam UMC, Amsterdam, Netherlands. ^16^Department of Clinical Chemistry, Amsterdam UMC, Amsterdam, Netherlands. ^17^Department of Clinical Epidemiology, Biostatistics and Bioinformatics, Amsterdam UMC, Amsterdam, Netherlands. ^18^Department of Neurology, Amsterdam UMC, Amsterdam, Netherlands.

*Leader of the AMC Consortium.

## COVID Human Genetic Effort

Laurent Abel^1^, Alessandro Aiuti^2^, Saleh Al Muhsen^3^, Fahd Al-Mulla^4^, Mark S. Anderson^5^, Andrés Augusto Arias^6^, Hagit Baris Feldman^7^, Dusan Bogunovic^8^, Alexandre Bolze^9^, Anastasiia Bondarenko^10^, Ahmed A. Bousfiha^11^, Petter Brodin^12^, Yenan Bryceson^12^, Carlos D. Bustamante^13^, Manish Butte^14^, Giorgio Casari^15^, Samya Chakravorty^16^, John Christodoulou^17^, Elizabeth Cirulli^9^, Antonio Condino-Neto^18^, Megan A. Cooper^19^, Clifton L. Dalgard^20^, Alessia David^21^, Joseph L. DeRisi^22^, Murkesh Desai^23^, Beth A. Drolet^24^, Sara Espinosa^25^, Jacques Fellay^26^, Carlos Flores^27^, Jose Luis Franco^28^, Peter K. Gregersen^29^, Filomeen Haerynck^30^, David Hagin^31^, Rabih Halwani​^32^, Jim Heath^33^, Sarah E. Henrickson^34^, Elena Hsieh^35^, Kohsuke Imai^36^, Yuval Itan^8^, Timokratis Karamitros^37^, Kai Kisand^38^, Cheng-Lung Ku^39^, Yu-Lung Lau^40^, Yun Ling^41^, Carrie L. Lucas^42^, Tom Maniatis^43^, Davoud Mansouri^44^, Laszlo Marodi^45^, Isabelle Meyts^46^, Joshua Milner^47^, Kristina Mironska^48^, Trine Mogensen^49^, Tomohiro Morio^50^, Lisa FP. Ng^51^, Luigi D. Notarangelo^52^, Antonio Novelli^53^, Giuseppe Novelli^54^, Cliona O’Farrelly^55^, Satoshi Okada^56^, Tayfun Ozcelik^57^, Rebeca Perez de Diego^58^, Anna M. Planas^59^, Carolina Prando^60^, Aurora Pujol^61^, Lluis Quintana-Murci^62^, Laurent Renia^63^, Alessandra Renieri^64^, Carlos Rodríguez-Gallego^65^, Vanessa Sancho-Shimizu^66^, Vijay Sankaran^67^, Kelly Schiabor Barrett^9^, Mohammed Shahrooei^68^, Andrew Snow^69^, Pere Soler-Palacín^70^, András N. Spaan^71^, Stuart Tangye^72^, Stuart Turvey^73^, Furkan Uddin^74^, Mohammed J. Uddin^75^, Diederik van de Beek^76^, Sara E. Vazquez^77^, Donald C. Vinh^78^, Horst von Bernuth^79^, Nicole Washington^9^, Pawel Zawadzki^80^, Helen C. Su^52^, Jean-Laurent Casanova^81^

^1^INSERM U1163, University of Paris, Imagine Institute, Paris, France. ^2^San Raffaele Telethon Institute for Gene Therapy, IRCCS Ospedale San Raffaele, Milan, Italy. ^3^King Saud University, Riyadh, Saudi Arabia. ^4^Kuwait University, Kuwait City, Kuwait. ^5^University of California, San Francisco, San Francisco, CA, USA. ^6^Universidad de Antioquia, Group of Primary Immunodeficiencies, Antioquia, Colombia. ^7^The Genetics Institute, Tel Aviv Sourasky Medical Center and Sackler Faculty of Medicine, Tel Aviv University, Tel Aviv, Israel. ^8^Icahn School of Medicine at Mount Sinai, New York, NY, USA. ^9^Helix, San Mateo, CA, USA. ^10^Shupyk National Medical Academy for Postgraduate Education, Kiev, Ukraine. ^11^Clinical Immunology Unit, Pediatric Infectious Disease Departement, Faculty of Medicine and Pharmacy, Averroes University Hospital; LICIA Laboratoire d’Immunologie Clinique, d’Inflammation et d’Allergie, Hassann Ii University, Casablanca, Morocco. ^12^Karolinska Institute, Stockholm, Sweden. ^13^Stanford University, Stanford, CA, USA. ^14^University of California, Los Angeles, CA, USA. ^15^Medical Genetics, IRCCS Ospedale San Raffaele, Milan, Italy. ^16^Emory University Department of Pediatrics and Children’s Healthcare of Atlanta, Atlanta, GA, USA. ^17^Murdoch Children’s Research Institute, Victoria, Australia. ^18^University of São Paulo, São Paulo, Brazil. ^19^Washington University School of Medicine, St. Louis, MO, USA. ^20^The American Genome Center; Uniformed Services University of the Health Sciences, Bethesda, MD, USA. ^21^Centre for Bioinformatics and System Biology, Department of Life Sciences, Imperial College London, South Kensington Campus, London, UK. ^22^University of California, San Francisco, CA, USA; Chan Zuckerberg Biohub, San Francisco, CA, USA. ^23^Bai Jerbai Wadia Hospital for Children, Mumbai, India. ^24^School of Medicine and Public Health, University of Wisconsin, Madison, WI, USA. ^25^Instituto Nacional de Pediatria (National Institute of Pediatrics), Mexico City, Mexico. ^26^Swiss Federal Institute of Technology Lausanne, Lausanne, Switzerland. ^27^Research Unit, Hospital Universitario Nuestra Señora de Candelaria, Canarian Health System, Santa Cruz de Tenerife, Spain. ^28^University of Antioquia, Medellín, Colombia. ^29^Feinstein Institute for Medical Research, Northwell Health USA, Manhasset, NY, USA. ^30^Department of Paediatric Immunology and Pulmonology, Centre for Primary Immunodeficiency Ghent (CPIG), PID Research Lab, Jeffrey Modell Diagnosis and Research Centre, Ghent University Hospital, Edegem, Belgium. ^31^The Genetics Institute, Tel Aviv Sourasky Medical Center, Tel Aviv, Israel. ^32^Sharjah Institute of Medical Research, College of Medicine, University of Sharjah, Sharjah, UAE. ^33^Institute for Systems Biology, Seattle, WA, USA. ^34^Children’s Hospital of Philadelphia, Philadelphia, PA, USA. ^35^Anschutz Medical Campus, Aurora, CO, USA. ^36^Riken, Tokyo, Japan. ^37^Hellenic Pasteur Institute, Athens, Greece. ^38^University of Tartu, Tartu, Estonia. ^39^Chang Gung University, Taoyuan County, Taiwan. ^40^The University of Hong Kong, Hong Kong, China. ^41^Shanghai Public Health Clinical Center, Fudan University, Shanghai, China. ^42^Yale School of Medicine, New Haven, CT, USA. ^43^New York Genome Center, New York, NY, USA. ^44^Shahid Beheshti University of Medical Sciences, Tehran, Iran. ^45^Semmelweis University Budapest, Budapest, Hungary. ^46^KU Leuven, Department of Immunology, Microbiology and Transplantation, Leuven, Belgium. ^47^Columbia University Medical Center, New York, NY, USA. ^48^University Clinic for Children’s Diseases, Skopje, North Macedonia. ^49^Aarhus University, Aarhus, Denmark. ^50^Tokyo Medical & Dental University Hospital, Tokyo, Japan. ^51^Singapore Immunology Network, Singapore. ^52^National Institute of Allergy and Infectious Diseases, National Institutes of Health, Bethesda, MD, USA. ^53^Bambino Gesù Children’s Hospital, Rome, Italy. ^54^Department of Biomedicine and Prevention, University of Rome “Tor Vergata,” Rome, Italy. ^55^Trinity College, Dublin, Ireland. ^56^Hiroshima University, Hiroshima, Japan. ^57^Bilkent University, Ankara, Turkey. ^58^Laboratory of Immunogenetics of Human Diseases, Innate Immunity Group, IdiPAZ Institute for Health Research, La Paz Hospital, Madrid, Spain. ^59^IIBB-CSIC, IDIBAPS, Barcelona, Spain. ^60^Faculdades Pequeno Príncipe e Instituto de Pesquisa Pelé Pequeno Príncipe, Curitiba, Brazil. ^61^Neurometabolic Diseases Laboratory, IDIBELL–Hospital Duran I Reynals; Catalan Institution for Research and Advanced Studies (ICREA); CIBERER U759, ISCiii Madrid Spain, Barcelona, Spain. ^62^Institut Pasteur (CNRS UMR2000) and Collège de France, Paris, France. ^63^Infectious Diseases Horizontal Technology Center and Singapore Immunology Network, Agency for Science Technology (A*STAR), Singapore. ^64^Medical Genetics, University of Siena, Siena, Italy; Genetica Medica, Azienda Ospedaliero-Universitaria Senese, Italy; GEN-COVID Multicenter Study. ^65^Hospital Universitario de Gran Canaria Dr. Negrín, Canarian Health System, Canary Islands, Spain. ^66^Imperial College London, London, UK. ^67^Boston Children’s Hospital, Harvard Medical School, Boston, MA, USA. ^68^Saeed Pathobiology and Genetic Lab, Tehran, Iran. ^69^Uniformed Services University of the Health Sciences, Bethesda, MD, USA. ^70^Hospital Universitari Vall d’Hebron, Barcelona, Spain. ^71^University Medical Center Utrecht, Amsterdam, The Netherlands. ^72^Garvan Institute of Medical Research, Sydney, Australia. ^73^The University of British Columbia, Vancouver, Canada. ^74^Holy Family Red Crescent Medical College; Centre for Precision Therapeutics, NeuroGen Children’s Healthcare; Genetics and Genomic Medicine Centre, NeuroGen Children’s Healthcare, Dhaka, Bangladesh. ^75^Mohammed Bin Rashid University of Medicine and Health Sciences, College of Medicine, Dubai, UAE; The Centre for Applied Genomics, Department of Genetics and Genome Biology, The Hospital for Sick Children, Toronto, Ontario, Canada. ^76^Amsterdam UMC, University of Amsterdam, Department of Neurology, Amsterdam Neuroscience, Amsterdam, The Netherlands. ^77^University of California, San Francisco, CA, USA. ^78^McGill University Health Centre, Montreal, Canada. ^79^Charité–Berlin University Hospital Center, Berlin, Germany. ^80^Molecular Biophysics Division, Faculty of Physics, A. Mickiewicz University, Uniwersytetu Poznanskiego 2, Poznań, Poland. ^81^Rockefeller University, Howard Hughes Medical Institute, Necker Hospital, New York, NY, USA.

*Leaders of the COVID Human Genetic Effort.

## NIAID-USUHS/TAGC COVID Immunity Group

Huie Jing^1,2^, Wesley Tung^1,2^, Christopher R. Luthers^3^, Bradly M. Bauman^3^, Samantha Shafer^2,4^, Lixin Zheng^2,4^, Zinan Zhang^2,4^, Satoshi Kubo^2,4^, Samuel D. Chauvin^2,4^, Kazuyuki Meguro^1,2^, Elana Shaw^1,2^, Michael Lenardo^2,4^, Justin Lack^5^, Eric Karlins^6^, Daniel M. Hupalo^7^, John Rosenberger^7^, Gauthaman Sukumar^7^, Matthew D. Wilkerson^7^, Xijun Zhang^7^

^1^Laboratory of Clinical Immunology and Microbiology, Division of Intramural Research, NIAID, NIH, Bethesda, MD, USA. ^2^NIAID Clinical Genomics Program, National Institutes of Health, Bethesda, MD, USA. ^3^Department of Pharmacology & Molecular Therapeutics, Uniformed Services University of the Health Sciences, Bethesda, MD, USA. ^4^Laboratory of Immune System Biology, Division of Intramural Research, NIAID, NIH, Bethesda, MD, USA. ^5^NIAID Collaborative Bioinformatics Resource, Frederick National Laboratory for Cancer Research, Leidos Biomedical Research, Inc., Frederick, MD, USA. ^6^Bioinformatics and Computational Biosciences Branch, Office of Cyber Infrastructure and Computational Biology, NIAID, NIH, Bethesda, MD, USA. ^7^The American Genome Center, Uniformed Services University of the Health Sciences, Bethesda, MD, USA.

## Supplementary Materials

science.sciencemag.org/content/370/6515/eabd4570/suppl/DC1 Materials and Methods Figs. S1 to S11 Tables S1 and S2 References (*42* and *43*) MDAR Reproducibility Checklist

## Appendix

View/request a protocol for this paper from *Bio-protocol*.
